# TNFSF13 Is a Novel Onco-Inflammatory Marker and Correlates With Immune Infiltration in Gliomas

**DOI:** 10.3389/fimmu.2021.713757

**Published:** 2021-10-12

**Authors:** Rui Chen, Xinxing Wang, Ziyu Dai, Zeyu Wang, Wantao Wu, Zhengang Hu, Xun Zhang, Zhixiong Liu, Hao Zhang, Quan Cheng

**Affiliations:** ^1^Department of Neurosurgery, The Affiliated Nanhua Hospital, Hengyang Medical School, University of South China, Hengyang, China; ^2^Department of Orthopedics, The Third Xiangya Hospital, Central South University, Changsha, China; ^3^Department of Neurosurgery, Xiangya Hospital, Central South University, Changsha, China; ^4^Department of Oncology, Xiangya Hospital, Central South University, Changsha, China; ^5^National Clinical Research Center for Geriatric Disorders, Xiangya Hospital, Central South University, Changsha, China; ^6^Department of Clinical Pharmacology, Xiangya Hospital, Central South University, Changsha, China

**Keywords:** TNFSF13, gliomas, immune infiltration, prognostic marker, inflammation

## Abstract

Existing therapeutic strategies for gliomas are restricted; hence, exploration for novel diagnostic indicator and treatment is essential. Here, we performed bioinformatic analyses for TNFSF13 (also known as APRIL), a proliferation-inducing ligand of the tumor necrosis factor (TNF) superfamily, aiming to assess its potential for predicting glioma patient’s prognosis and targeted therapy. TNFSF13 expression was upregulated in the increase of tumor grades based on Xiangya cohort. In high TNFSF13 gliomas, somatic mutation was proved to correlate with amplification of EGFR and deletion of CDKN2A; while mutation of IDH1 was more frequently observed in low TNFSF13 group. We also confirmed the positive correlation between TNFSF13 and infiltrating immune and stromal cells in glioma microenvironment. Further, TNFSF13 was found to be involved in immunosuppression *via* diverse immunoregulation pathways and was associated with other immune checkpoints and inflammation. Single-cell sequencing revealed an abundant expression of TNFSF13 in neoplastic cells and M2 macrophages, which TNFSF13 might potentially regulate the cell communication *via* IL-8, C3, and CD44. Lastly, TNFSF13 mediated the activities of transcription factors including FOXO3, MEIS2, and IRF8. Our analyses demonstrated the relevance between TNFSF13 and glioma progress and indicated the potential of TNFSF13 as a novel diagnostic onco-inflammatory biomarker and immunotherapy target of gliomas.

## Introduction

Gliomas are the most prevalent malignant tumors of the adult’s central nervous system (CNS), in which glioblastoma (GBM) is the most malignant type. GBM patients experience a short median survival time about 15 months from primary diagnosis, in spite of the advancement in integrated therapeutic schedules, such as the classical neurosurgical resection with adjuvant radiotherapy, and alkylating agent temozolomide chemotherapy (1–4). The poor survival of GBM patients can be attributed to infiltrative property in the progress of tumor invasion (5), microscopic propagation of GBM cells (6), resistance to therapeutic drugs, and tumor recurrence (3).

The significant advancement in revealing the molecular mechanisms in the progression of GBM has promoted the exploration of novel directions in the treatment of GBM (7, 8). Unfortunately, many molecular-targeted therapeutic methods failed to acquire a favorable clinical outcome, despite the promising results in preclinical models (9). Relevant researches have ascribed the dismal clinical outcome to the multiformity of cells and evolution in GBM microenvironment, blood-brain barrier (BBB), and resistance to therapeutic drugs (10–13). Considering the poor outcome of GBM patients under conventional therapies with surgery and adjuvant chemoradiotherapy, immunotherapy emerges as a novel promising therapeutic strategy that activates immune system to destroy cancer cells (14, 15). During the whole dynamic process of immune activity, immune checkpoints, defined as a group of bio-effectual molecules, have been proved to play a dominant role in the immune response in GBM by modulating T cell activity and making the immune system self-tolerable (16, 17). Accumulating evidences have revealed that high expression of many traditional checkpoints, such as PDCD1LG2, HAVCR2, and CTLA4, mediated suppression of immune response and ultimately led to immune evasion in GBM (18–25). Accordingly, further investigation on immune checkpoint inhibitors would promote novel and effective treatment strategies in prospect.

TNFSF13 (also known as APRIL), a proliferation-inducing ligand of the tumor necrosis factor (TNF) superfamily, has been identified as a promising molecular target to overcome immunosuppression. Notably, TNFSF13 has been demonstrated to exert a vital role in lymphocyte maturation, physiological activities, and pathological activities, such as neoplasia (26). TNFSF13 was proved to interact with two receptors: B cell maturation antigen (BCMA), which germanely combined with TNFSF13 to induce tumor cell invasion and immunosuppression (27, 28); the other one is transmembrane activator and calcium modulator and cyclophilin ligand interactor (TACI), which have both positive and negative regulation to B cell activity (29). TNFSF13 expression has been explored in different tumors (30). In breast cancer, TNFSF13 expression is associated with breast cancer classification (31). In multiple myeloma (MM), TNFSF13 facilitates MM cell survival by increasing regulatory B cells and T cells *via* TACI in bone marrow microenvironment (32). Moreover, one recent study has demonstrated that TNFSF13 correlated with human triple negative carcinomas and induced tumor cell proliferation, which also confirmed the role of TNFSF13 in tumor aggressiveness (33). The above findings support the speculation that TNFSF13 might be involved in the regulation of immune activities and tumor invasion of gliomas.

To explore and confirm the role of TNFSF13 in promoting the progression of gliomas, we performed several analyses *via* public available databases, and the corresponding glioma patient samples with TNFSF13 expression data were collected from TCGA and CGGA datasets. Our results suggested that TNFSF13 expression was upregulated in GBM and associated with immunosuppression, which supported the claim that TNFSF13 could be a potential diagnostic marker and treatment target in the clinical management of glioma patients.

## Materials and Methods

### Data Collection

All transcriptomic data of TNFSF13 in low-grade glioma (LGG) and GBM cohorts were collected from the Cancer Genome Atlas (TCGA) and Chinese Glioma Genome Atlas (CGGA) datasets, among which 672 cases of TCGA were obtained from UCSC Xena (https://xenabrowser.net/) and 1,013 cases of CGGA were from CGGA website (http://www.cgga.org.cn/). RNA-seq of TNFSF13 for diverse intratumor anatomic structures in GBM was from Ivy Glioblastoma Atlas Project (http://glioblastoma.alleninstitute.org/). Single-cell expression matrices were obtained from the Gene Expression Omnibus (GEO; https://www.ncbi.nlm.nih.gov/) GSE84465, which performed single-cell/nuclei RNA-sequencing of four GBM samples (34). A total of 3,589 cells (1,246 cells within periphery normal tissue, 2,340 cells within tumor tissue) were identified in the four scRNA sequencing samples, and 2,340 tumor cells were used for analysis.

### Genomic Alteration

Cases with somatic mutations and somatic copy number alternations (CNAs) were collected from TCGA datasets. CNAs correlated with TNFSF13 expression, and the threshold copy number at alteration peaks were analyzed by GISTIC 2.0 (https://gatkforums.broadinstitute.org) (35). In addition, we employed cBioPortal (36) (http://www.cbioportal.org) to analyze the mutation frequency of TNFSF13 in TCGA database.

### Immune Infiltration

ESTIMATE (Estimation of Stromal and Immune cells in Malignant Tumor tissues using Expression) algorithm generated three scores, among which immune score was the reflection of immune cell infiltration level, stromal score was the reflection of stromal cell presence level, and estimate score was to evaluate the purity of tumor. Tumor Immune Estimation Resource 2.0 (TIMER2.0; http://timer.cistrome.org/) web server (37) was used for comprehensive analysis of immune infiltrating cells across diverse cancer types.

### Functional Annotation

Gene ontology (GO) analysis was performed with gene set variation analysis (GSVA) using the R package GSVA (38) and revealed the differential expression of multiple immune-related signaling pathways in pan-glioma and GBM. Spearman correlation analysis performed with the expression values of TNFSF13 picked out events with low p value (<0.05) and high correlation coefficient (>0.4).

### Single-Cell RNA Analysis

We processed the single-cell data expression matrix with the R package Seurat V3.1.2. At first, we used “NormalizeData” to normalize the gene expression data, then “FindVariableGenes” was applied to confirm 2,000 highly variable genes (HVGs). Afterwards, we used “FindIntegrationAnchors” and “Integratedata” to merge four glioma sample data (39). After performing principal component analysis (PCA) with “RunPCA,” we built a K-nearest neighbor graph *via* the “FindNeighbors” function and combined cells together in a highest resolution with “FindClusters.” Ultimately, “UMAP” was used for visualization, and we performed “Single R” R package for cell annotation. “FeaturePlot” and “VlnPlot” were used to visualize TNFSF13 expression. Single-cell pseudotime trajectories were reconstructed and analyzed with package “Monocle” (40). In short, cells were dimensionality-reduced and arranged into a trajectory, which was segmented into five states with two branch points. Additionally, GSEA for TNFSF13 in each state was constructed. Gene ontology (GO) enrichment analysis and pathway analysis based on Kyoto Encyclopedia of Genes and Genomes (KEGG) were also performed. Cell communication patterns in the tumor microenvironment of GBM were explored using the R package iTALK. In addition, connection specificity index (CSI) of regulon was the evaluation index for the correlation among various regulons. High index of regulon represents a high capability of regulating downstream genes and influencing biological process of malignant cells. Regulon modules were distinguished according to CSI of regulon (41). Then we conducted gene regulatory network analysis based on DNA motif and co-expression of regulons using the R package SCENIC (42). Activity scores of all regulons in neoplastic cells were evaluated using the R package AUCell (43).

### Immunohistochemistry

Written informed consent was obtained from all patients, and the study methodologies were approved by the Ethics Committee of Xiangya Hospital, Central South University. The IHC tissue samples are provided in **Supplementary Table S1**. The detailed protocol for the IHC experiment was as previously described (44). Slides were blocked with 5% BSA and incubated with primary antibody (rabbit polyclonal anti-TNFSF13 antibody, 1:500; Proteintech; Wuhan, China) at 4°C overnight.

### Statistical Analysis

The Kaplan-Meier survival curve was generated to evaluate survival distributions, and the log-rank test was to detect the statistical significance. We used the Student’s t-test for normally distributed variables and the Wilcoxon test for nonnormally distributed variables to compare the expression levels of TNFSF13 between two groups regarding different pathological factors. For multiple groups, one-way analysis of variance (ANOVA) was used to compare the expression levels of TNFSF13 among groups. Pearson correlation and spearman correlation were used to evaluate the linear relevance between continuous variables, and the gene expression was visualized by taking advantage of “RaincloudPlot”, “VlnPlot” and “FeaturePlot.” The R package Survival was used in Cox regression analysis. All statistical analyses were accomplished with R project (version 3.6.3, https://www.r-project.org/). P-value <0.05 was regarded as statistically significant, and all tests were two-sided.

## Results

### Correlations of TNFSF13 Expression With Clinical and Molecular Traits in Gliomas

**Figure 1:**
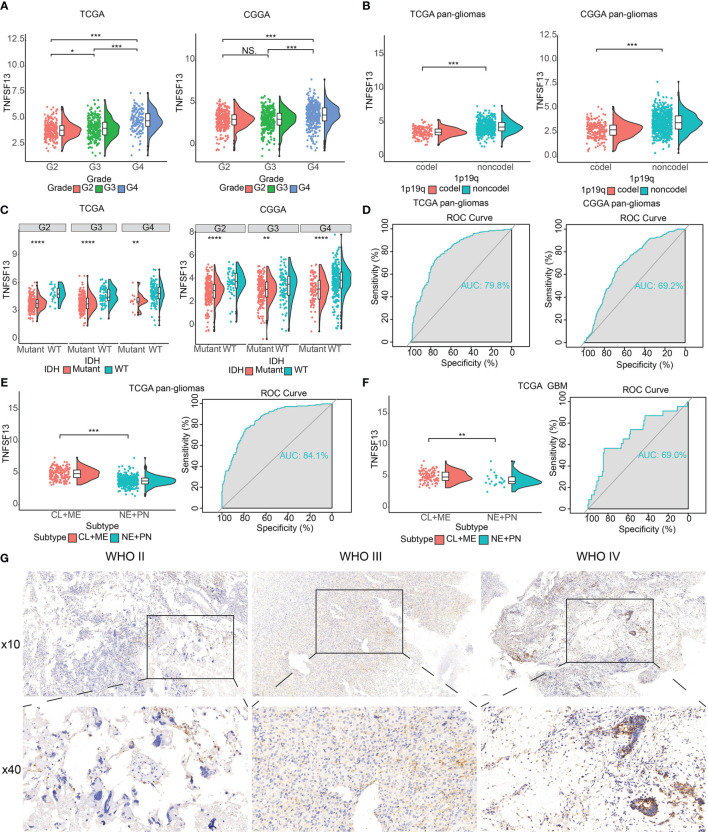
The clinical and molecular characteristics in associations with TNFSF13 expression. **(A)** The expression levels of TNFSF13 in different WHO grades from TCGA and CGGA datasets. **(B)** TNFSF13 expression increased in 1p/19q non-codeletion glioma cases. **(C)** TNFSF13 expression levels in different IDH states from TCGA and CGGA datasets. **(D)** Receiver operating characteristic (ROC) curve showed 79.8 and 69.2% sensitivity and specificity of TNFSF13 to predict IDH wild-type gliomas from TCGA and CGGA, respectively. **(E)** The TNFSF13 expression levels in pan-gliomas of different molecular subtypes from TCGA dataset. ROC curves to assess sensitivity and specificity of TNFSF13 expression to predict more invasive subtype gliomas. **(F)** The TNFSF13 expression levels in GBM of different molecular subtypes from TCGA dataset. ROC curve showed 80.4% sensitivity and specificity of TNFSF13 expression to predict more invasive subtype gliomas. **(G)** Typical images of IHC staining for TNFSF13 in different pathological grades of glioma samples from Xiangya cohort. NS, not statistically significant, *P <.05, **P <.01, ***P <.001, ****P <.0001.

We found a relatively high correlation between TNFSF13 and U-251 MG cell lineage (**Supplementary Figure S1A**) according to the data from TCGA and CGGA datasets, and TNFSF13 expression was highly associated with various tumors, such as GBM, although there was no significant enhanced expression of TNFSF13 in low-grade glioma (LGG) (**Supplementary Figure S1B**). Then we evaluated TNFSF13 expression based on World Health Organization (WHO) classification and histology, which showed the expression of TNFSF13 was enhanced in GBM (WHO grade IV) compared with LGG (WHO grade II and III) cases (**Figure 1A**), and TNFSF13 level was upregulated in higher histopathologic malignancies (**Supplementary Figure S1C**). Meanwhile, some genetic alterations correlated with heterogeneous tumor histology were detected, including 1p/19q codeletion and IDH mutation (45), TNFSF13 expression was found amplified in 1p/19q non-codeletion glioma samples (**Figure 1B**), and compared to IDH mutation cases, the expression of TNFSF13 enhanced in IDH wild-type gliomas of all WHO grades (**Figure 1C**). Moreover, Receiver operating characteristic (ROC) curve was used to assess the sensitivity and specificity of TNFSF13 expression to distinguish IDH wild-type gliomas from normal tissue. Expectedly, the area under the curve (AUC) were 79.8% in TCGA dataset and 69.2% in CGGA dataset (**Figure 1D**), which suggested that TNFSF13 might be a potential biomarker in the developing progress of glioma (46). Epigenetic alterations such as DNA methylation would also promote carcinogenesis (47). Seventeen methylation probes were identified for TNFSF13 from TCGA, which all of them exhibited remarkable negative association with expression of TNFSF13, and most of the association was statistically significant (**Supplementary Figures S2**, **S3**). We subsequently investigated TNFSF13 expression in specific tumor anatomic structure based on Ivy Glioblastoma Atlas Project, and found high expression of TNFSF13 was enriched in hyperplastic blood vessels, leading edge and peri-necrotic zone (**Supplementary Figure S1D**), which play a crucial role in the development of tumors.

According to transcriptomic and genomic dimensions, molecular classification was made to predict diverse patient outcomes (48). Consequently, we evaluated the expression of TNFSF13 in different molecular subtypes, including classical (CL), mesenchymal (ME), pro-neural (PN), and neural (NE), in which CL and ME subtypes behave more aggressively compared with PN or NE subtypes (49). As shown from TCGA dataset, TNFSF13 was surprisingly upregulated in CL and ME subtypes contrasted with other subtypes among pan-glioma cases, as well as GBM cases. Furthermore, the area under the curve (AUC) of TNFSF13 was 84.1% in pan-glioma cohort and 69.0% in GBM cohort (**Figures 1E, F**). These results indicated that TNFSF13 might serve as an effective biomarker for CL and ME subtypes.

Moreover, to identify the upregulation of TNFSF13 expression at the protein level, we conducted the IHC staining for TNFSF13 based on the 40 samples from Xiangya cohort (**Figure 1G**). The images revealed an enhanced expression of TNFSF13 in the increase of pathological grades of glioma samples. In conclusion, these results demonstrated that TNFSF13 might be a predictive biomarker for more aggressive tumor subtypes and play a crucial part in the progression of glioma.

### TNFSF13 Expression Associates With Poor Survival in Glioma Patients

Subsequently, we evaluated the prognostic value of TNFSF13 expression in human gliomas by Kaplan-Meier analysis. Based on TCGA and CGGA datasets, analysis of pan-glioma cohorts revealed that patients with higher expression of TNFSF13 exhibited dramatically poorer overall survival (OS) than patients with lower expression of TNFSF13, and similar correlations were noticed in LGG and GBM cohorts (**Supplementary Figures S4A, B**). Additionally, higher TNFSF13 expression was relevant to worse progression-free survival (PFS) and disease-specific survival (DSS) in pan-gliomas, LGG, and GBM cohorts (**Supplementary Figure S5**). Subgroup analyses were made to evaluate the prognostic value of TNFSF13 in different tumors, and these results revealed that TNFSF13 was a hazardous prognostic indicator in 15 independent tumor cohorts including LGG and GBM, and TNFSF13 was also a favorable prognostic indicator in 18 independent tumor cohorts according to OS (**Supplementary Figure S4C**). Analysis in regard to DSS showed similar results about the prognostic value of TNFSF13 (**Supplementary Figure S4D**). Consequently, the expression of TNFSF13 was relevant to worse prognosis in glioma patients.

In addition, we investigated the prognostic value of TNFSF13 in other cancers according to OS and DDS. Then diverse correlations were revealed between TNFSF13 and various tumors including adrenocortical carcinoma (ACC), cholangiocarcinoma (CHOL), kidney renal clear cell carcinoma (KIRC), kidney renal papillary cell carcinoma (KIRP), lung adenocarcinoma (LUAD), lung squamous cell carcinoma (LUSC), mesothelioma (MESO), pancreatic adenocarcinoma (PAAD), sarcoma (SARC), skin cutaneous melanoma (SKCM), uterine carcinosarcoma (UCS), uveal melanoma (UVM) (**Supplementary Figures S6**, **S7**).

To better predict clinical prognosis for patients, we further built a prognostic nomogram model with OS and five independent risk factors (the expression level of TNFSF13, age, grades of gliomas, mutation of IDH, and 1p19q codeletion) based on previous investigation, in which high score represented worse survival overcome (**Supplementary Figure S8A**). Based on clinical 1-year OS, 3-year OS, 4-year OS, and 5-year OS in TCGA (**Supplementary Figure S8B**) and 4-year OS in CGGA (**Supplementary Figure S8E**), calibration plots revealed that the nomograms were favorable to predict patient survival according to a conceptual model. We next assessed the statistical significance of diverse survival overcome with high-risk and low-risk patients from TCGA and CGGA datasets (**Supplementary Figures S8C, F**). ROC curve (AUC>=0.872 in TCGA, AUC>=0.771 in CGGA) further revealed that expression of TNFSF13 might be an idea predictor for the survival outcome of glioma patients (**Supplementary Figures S8D, G**).

### TNFSF13 Expression Is Relevant to Distinct Genomic Alterations

**Figure 2:**
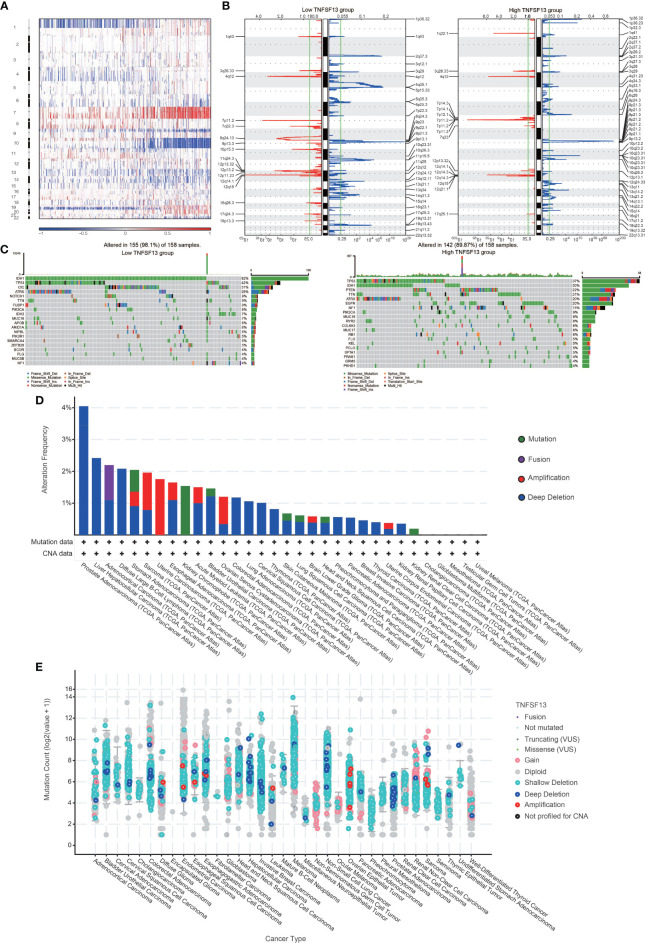
Distinct genomic profiles associated with TNFSF13 expression and integrative analysis of complex cancer genomics and clinical profiles using the cBioPortal. **(A)** The overall CNAs profile about high and low TNFSF13 expression. **(B)** Frequency of amplifications and deletions in gliomas with low and high TNFSF13 expression. Blue, deletion; red, amplification. **(C)** Detection of differential somatic mutations in gliomas. **(D)** Frequency of four alterations of TNFSF13 in different cancers. **(E)** TNFSF13 expression level of various alterations in 35 types of cancer.

To estimate the correlations between expression of TNFSF13 and genomic characteristics in gliomas, somatic mutations and copy number alternations (CNA) analysis were performed using TCGA database. An overall CNA profile was generated according to the comparison of high TNFSF13 expression cluster (n=158) *vs* low TNFSF13 expression cluster (n=158) (**Figures 2A, B**). In the high TNFSF13 expression cohort, typical genomic alterations in GBM-like amplification of chr7 and deletion of chr10 were observed (**Figure 2A**); while in the low TNFSF13 expression cohort, deletion of 1p and 19q, a distinguishing genomic feature of oligodendroglioma (50), was exhibited more frequently (**Figure 2B**). In high TNFSF13 expression cases, we observed amplification of genomic peaks, containing specific driver oncogenes including EGFR (7p11.2), PDGFRA (4q12), and CDK4 (12q14.1), accompanied by deleted peaks including tumor suppressor genes like CDKN2A and CDKN2B (9p21.3). Moreover, somatic mutation analysis based on TNFSF13 expression revealed high mutation frequency profiles including TP53 (37%), IDH1 (30%), PTEN (22%), TTN (21%), ATRX (20%), and EGFR (20%) in high TNFSF13 expression group (n=158), while mutation of IDH1 (82%), TP53 (42%), CIC (31%), and ATRX (25%) were more frequently noticed in low TNFSF13 expression group (n=158) (**Figure 2C**). Then we explored the mutation frequency of TNFSF13 in pan cancer from TCGA database based on cBioPortal, and prostate adenocarcinoma showed a relatively high mutation level with the TNFSF13 alteration frequency (4% approximately) (**Figures 2D, E**).

### TNFSF13 Is Correlated With Infiltrating Immune and Stromal Cells in Glioma Microenvironment

Previous molecular research reminded us that considerable cellular components forming tumors play a crucial part in tumor signal and cancer biology, such as infiltrated immune and stromal cells (51). Hence, an analysis to estimate the correlation between TNFSF13 and ESTIMATE scores was arranged. The results indicated that TNFSF13 expression was positively relevant to immune score, stromal score, and ESTIMATE score in pan-glioma group (**Supplementary Figure S9A**) with noticeable trends, and similar conclusion was observed in GBM group (**Supplementary Figure S9B**), which suggested a positive correlation between TNFSF13 expression level and infiltration of immune and stromal cells. To further explore particular cell categories related to TNFSF13 expression in tumor microenvironment, we performed a cell enrichment analysis to evaluate the correlation between TNFSF13 and 28 specific infiltrating cell types based on TCGA and CGGA pan-glioma datasets (52). In result, TNFSF13 expression was observed positively associated with various categories of infiltrating immune cell, including NK cells, monocytes, macrophages, CD8+ T effector memory cells (TEM), CD4+ TEM, MDSC, and regulatory T cells (Treg); however, negative correlation was found between TNFSF13 and type 2 T helper cell and memory B cells (**Supplementary Figures S9C, D**). In addition, we established a 10-immune cell lineage analysis to assess the enrichment of different immune cell lineages (53) based on pan-gliomas and GBM cohorts in TCGA and CGGA databases, and revealed the tight positive correlation between TNFSF13 expression and presence of multiple stromal cells, including neutrophils, monocytic lineage, and fibroblasts (**Supplementary Figures S10A, B**). In general, these data indicated high TNFSF13 expression might possess recruitment function for infiltrating immune and stromal cells into tumor microenvironment.

**Figure 3:**
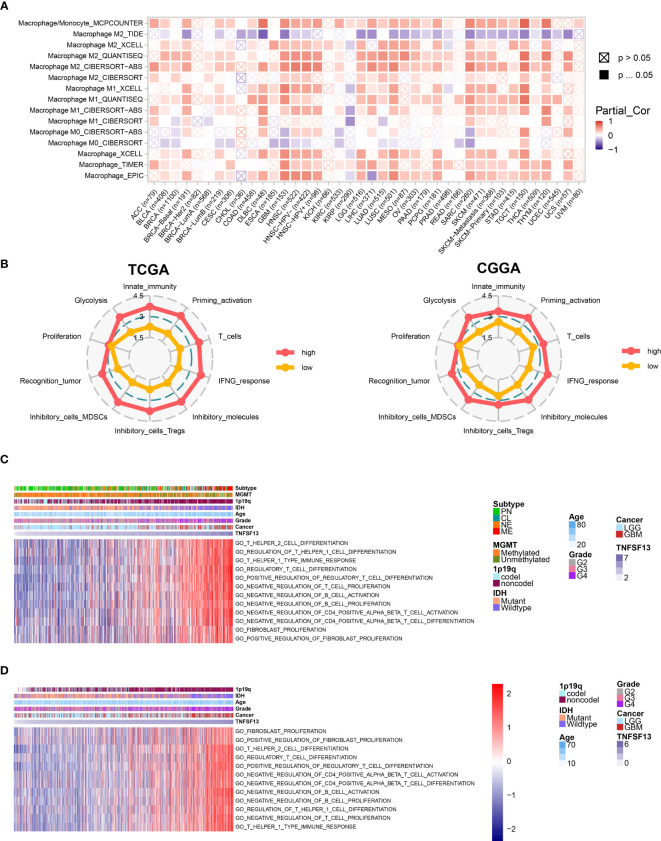
Correlation between TNFSF13 and immunologic functions in gliomas. **(A)** Immune infiltration assessment for pan-cancer samples regarding macrophage based on TIMER 2.0. Red represents high expression of macrophage; purple represents low expression of macrophage. **(B)** Ten immunogram scores to analyze the correlation between TNFSF13 expression and tumor-infiltrating immune cells in gliomas from TCGA cohort and CGGA cohort. Red represents high TNFSF13 expression; yellow represents low TNFSF13 expression. The relationship between TNFSF13 and T cell immunity in pan-gliomas from **(C)** TCGA and **(D)** CGGA datasets.

We subsequently assessed the macrophage infiltration in 40 solid cancers based on eight algorithms from TIMER 2.0 web server, and revealed a relatively high infiltration level of macrophages in several solid cancer types including GBM and LGG, while the correlation coefficients calculated by Spearman’s correlation analysis were relatively higher in GBM than LGG (**Figure 3A**). Besides, TNFSF13 was highly associated with T cells in pan-cancer samples (**Figure S10C**). Furthermore, we evaluated 10 immunogram scores for gliomas with different TNFSF13 expression level (**Figure 3B**), and the results indicated that high TNFSF13 cohort appeared higher scores in nine immunogram terms than low TNFSF13 cohort, and nine terms included glycolysis, innate immunity, priming activation, T cells, interferon γ (IFNG) response, inhibitory molecules, inhibitory cells Tregs (regulatory T cells), inhibitory cells MDSCs (myeloid-derived suppressor cells), and recognition of tumor cells. These were further evidence that TNFSF13 expression associated with immune response in gliomas, probably in a negative way.

In addition, previous several researches have reported that TNFSF13 might efficiently facilitate the immunotherapies depended on T cells (32, 54, 55). In order to verify whether TNFSF13 was relevant to T cell immunity, we performed GSVA analysis based on TCGA cohort, and the result indicated that TNFSF13 was negatively associated with T cell proliferation, CD4 positive alpha beta T cell activation, and CD4 positive alpha beta T cell differentiation. In contrast, TNFSF13 was positively relevant to regulatory T cell differentiation, regulation of T helper 1 cell differentiation, T helper 1 type immune response, and regulation of fibroblast proliferation (**Figure 3C**). Similar results were demonstrated in CGGA cohort (**Figure 3D**). Accordingly, TNFSF13 might contribute to the inhibition of T-cell based antitumor immune processes in gliomas.

### TNFSF13 Associated With Other Immune Checkpoint Molecules and Inflammatory Activities in Gliomas

**Figure 4:**
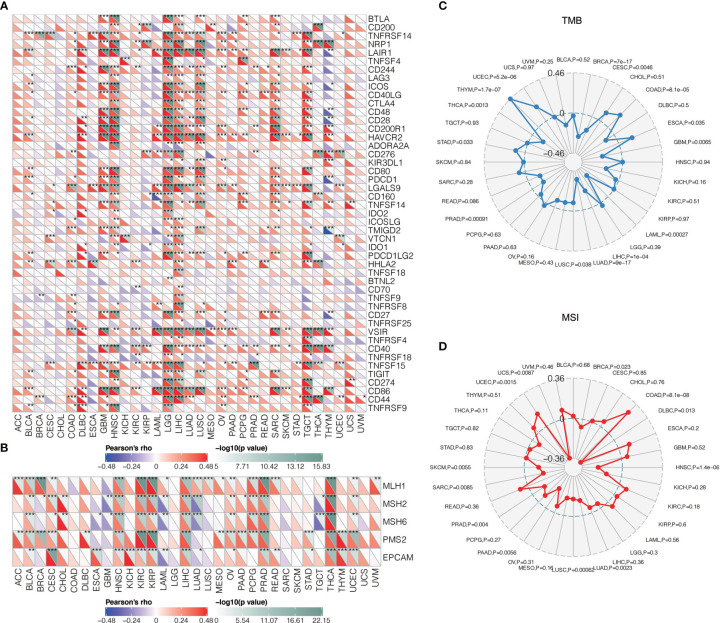
Correlations between TNFSF13 and immunogenic factors as well as tumorigenic factors. **(A)** The correlations between TNFSF13 and immune checkpoints in different cancers (*P < 0.05, **P < 0.01, ***P < 0.001). **(B)** The correlations between TNFSF13 and signature genes for MMR in different cancers (*P < 0.05, **P < 0.01, ***P < 0.001). **(C)** The correlations of TNFSF13 expression and TMB in different cancers. **(D)** The correlations of TNFSF13 expression and MSI in different cancers.

Given recent studies that showed a vital function of combination therapy with inhibiting immune checkpoints (56, 57), we performed a correlation analysis of several well-known immune checkpoint molecules to assess the relationship between TNFSF13 and them in gliomas. Based on TCGA and CGGA datasets, we observed a high and positive correlation between TNFSF13 and PDCD1LG2, HAVCR2, CTLA4, and other checkpoint molecules in pan-gliomas, LGG and GBM analysis, and TNFSF13 expression is tightly correlated with HAVCR2 in glioblastomas (**Supplementary Figures S11A, B**). These results demonstrated that the combination of TNFSF13 and other immune checkpoint molecules might serve as potential signaling pathways to contribute to the immunosuppression in gliomas. Besides, the pan-cancer correlations between TNFSF13 and immune checkpoints were shown in **Figure 4A**, which TNFSF13 positively correlated with the majority of the immune checkpoint molecules in most cancers.

Mismatch repair (MMR) has been critical in tumor progression during DNA replication and genetic recombination (58). DNA mismatch repair deficiency was closely linked to microsatellite instability (MSI) (59) that could contribute to the accumulation of tumor mutation burden (TMB) (60). Mismatch repair, MSI, TMB have all been proposed to be responsible for tumor growth and could efficiently predict immunotherapy responses (61). In our findings, TNFSF13 was positively associated with signature genes for MMR in most cancers (**Figure 4B**). In addition, the overall correlation between TNFSF13 and TMB, MSI in 32 cancers was remarkable (**Figures 4C, D**). Taken together, TNFSF13 could be a robust marker in predicting immunotherapy response.

In consideration of the relevance between TNFSF13 and the inflammatory response in gliomas revealed by our analysis, we subsequently investigated seven inflammatory activity signatures to clarify the function of TNFSF13 in the inflammatory response (62). Consequently, TNFSF13 expression was identified positively correlated with interferon, STAT1, MHC-I, MHC-II, HCK, and LCK, while negatively relevant to IgG metagene in pan-glioma analysis (**Supplementary Figures S11C, D**) and GBM alone (**Supplementary Figures S12A, B**) analysis in both TCGA and CGGA databases. These results demonstrated that TNFSF13 expression was upregulated in macrophage activation, T-cell-related signaling transduction, and antigen-presenting cells, while negatively associated with B-lymphocyte-related metagenes during immunosuppression in the progression of gliomas.

### Single-Cell Sequencing Investigated the Correlation Between TNFSF13 and Cell Clusters

**Figure 5:**
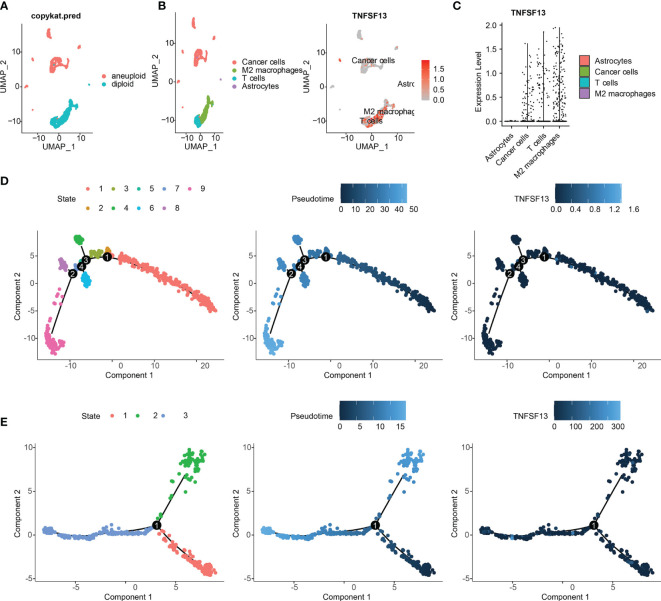
scRNA-seq results for TNFSF13 in GBM. **(A)** Tumor cells were defined as cells with aneuploid using R package copykat. **(B)** (*Left*) Cells were annotated into five clusters. (*Right*) Scatter plots of TNFSF13 expression in five clusters. Gray dots represent cells without TNFSF13. Red dots represent cell with TNFSF13 expression, and the shade of red represents expression level. **(C)** Violin plot of TNFSF13 expression distribution of five cell clusters. **(D)** Single-cell trajectory analysis of neoplastic cells reveals four main branches. Cells are colored based on states (*left*), pseudotime (*middle*), and TNFSF13 expression (*right*). **(E)** Single-cell trajectory analysis of macrophages reveals four branches. Cells are colored based on states (*left*), pseudotime (*middle*), and TNFSF13 expression (*right*).

To further evaluate the relevance between TNFSF13 and immune infiltrating, we performed scRNA-seq analysis to investigate the TNFSF13 expression level in different cell clusters in gliomas. Firstly, we performed “copykat” R package to identify tumor cells with aneuploid expression, and normal cells with diploid based on four glioma samples (**Figure 5A**). Afterwards, four cell clusters including cancer cells (also called neoplastic cells), M2 macrophages, astrocytes, and T cells were annotated and visualized; meanwhile, we showed the TNFSF13 expression in all clusters (**Figure 5B**). We further contrasted the expression level of TNFSF13 in each cluster and confirmed TNFSF13 was notably upregulated in M2 macrophages, T cells, and cancer cells (**Figure 5C**). Subsequently, we explored the Single-cell pseudotime trajectory analysis of neoplastic cells (**Figure 5D**) and M2 macrophages (**Figure 5E**). Aimed at neoplastic cells and M2 macrophages, we performed “Monocle” R package to reconstruct the pseudotime trajectory. In neoplastic cells, five main branches and four branch points were identified, and cells were separated into nine states. In M2 macrophages, three main branches and one branch point were identified, and cells were separated into three states. Then we observed relatively high TNFSF13 expression in state eight and state nine of neoplastic cells, while TNFSF13 slightly upregulated in state two and three of M2 macrophages.

**Figure 6:**
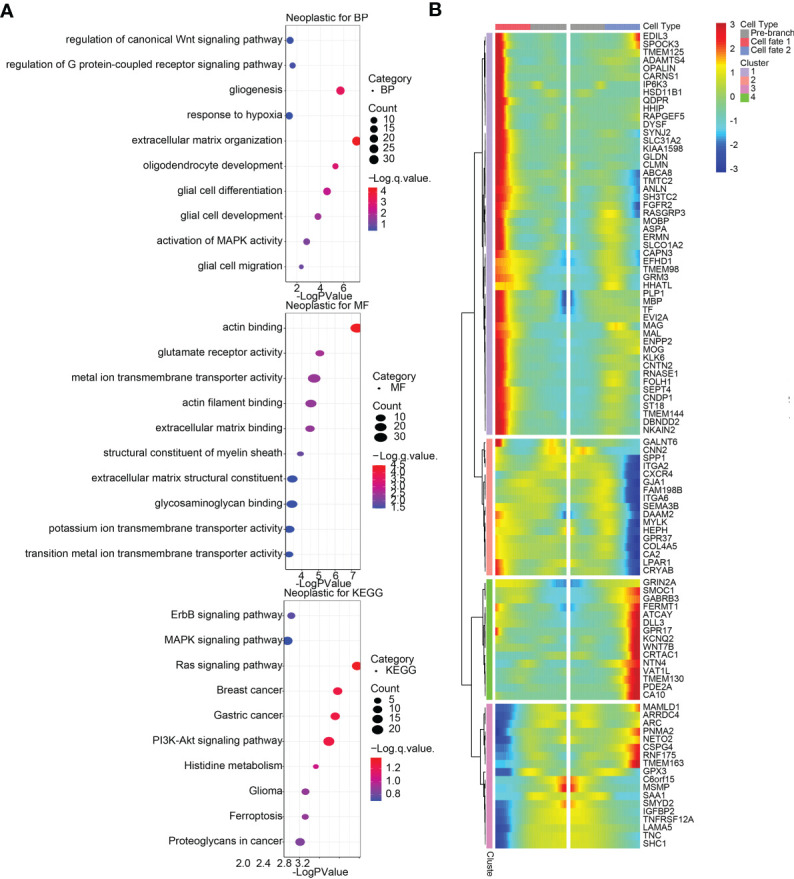
Differentially expressed genes (DEGs) and functional annotations of glioma neoplastic cells in single-cell pseudotime trajectories. **(A)** Biological processes (BP) and molecular functions (MF) from Gene Oncology (GO) enrichment analysis and Kyoto Encyclopedia of Genes and Genomes (KEGG) pathway analysis based on DEGs for branch point 2 in neoplastic cells. **(B)** Heatmap of top 100 DEGs with branch-dependent expression for branch point 2 in neoplastic cells.

Afterwards, we confirmed top 100 differentially expressed genes (DEGs) before and after branch point two in neoplastic cells (**Figure 6B**), among which top 12 genes were exhibited in **Supplementary Figure S14B**, and top six DEGs among five states in pseudotime trajectories of neoplastic cells were visualized in **Supplementary Figure S14A**. Additionally, we performed functional annotations including GO enrichment analysis and KEGG pathway analysis based on these DEGs (**Figure 6A**). In the same way, we identified top 100 DEGs with branch-dependent expression for branch point one in M2 macrophages (**Supplementary Figure S13B**), and functional annotations for these genes were performed (**Supplementary Figure S13A**). Top 12 genes were exhibited in **Supplementary Figure S14D**, and top six DEGs among five states in pseudotime trajectories of M2 macrophages were visualized in **Supplementary Figure S14C**. These still further testified that TNFSF13 expression was positively related to M2 macrophages and the progression of glioma.

**Figure 7:**
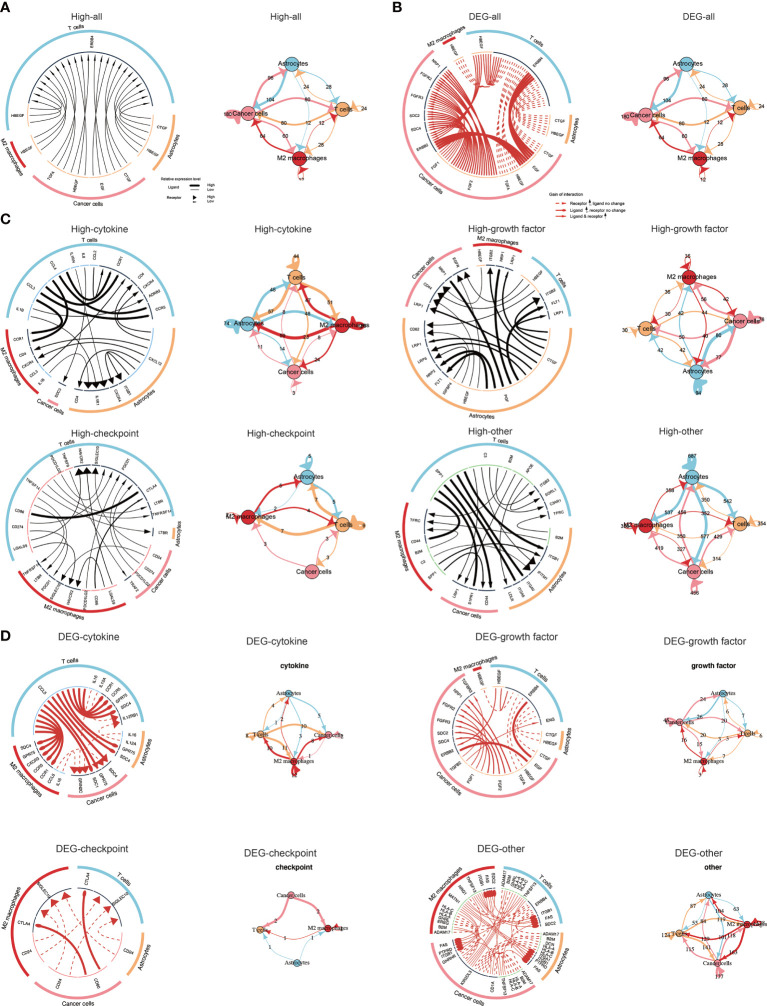
Visualization of cell communication. **(A)** Network plot and circos plot showing the total ligand-receptor interactions detected between each two different cell types and/or within the same cell type in high TNFSF13 group. **(B)** Network plot and circos plot showing the total ligand-receptor interactions detected between each two different cell types and/or within the same cell type based on the differentially expressed genes between high TNFSF13 group and low TNFSF13 group. **(C)** Network plot and circos plot showing the four types of ligand-receptor interactions detected between each two different cell types and/or within the same cell type in high TNFSF13 group. **(D)** Network plot and circos plot showing the four types of ligand-receptor interactions detected between each two different cell types and/or within the same cell type based on the differentially expressed genes between high TNFSF13 group and low TNFSF13 group.

Particularly, we explored ligand-receptor interactions in cells among those with high TNFSF13 expression (**Figure 7A**), and detailed four categories of intercellular communication were displayed in **Figure 7C**. Apparently, we observed strong interactions among cancer cells, T cells, and M2 macrophages, especially in checkpoint analysis (**Figure 7C**). In the meantime, we analyzed ligand-receptor interactions in cells based on differentially expressed genes between high TNFSF13 group and low TNFSF13 group (**Figure 7B**); more detailed classifications were displayed in **Figure 7D**. As expected, close interactions among cancer cells, T cells, and M2 macrophages were observed.

**Figure 8:**
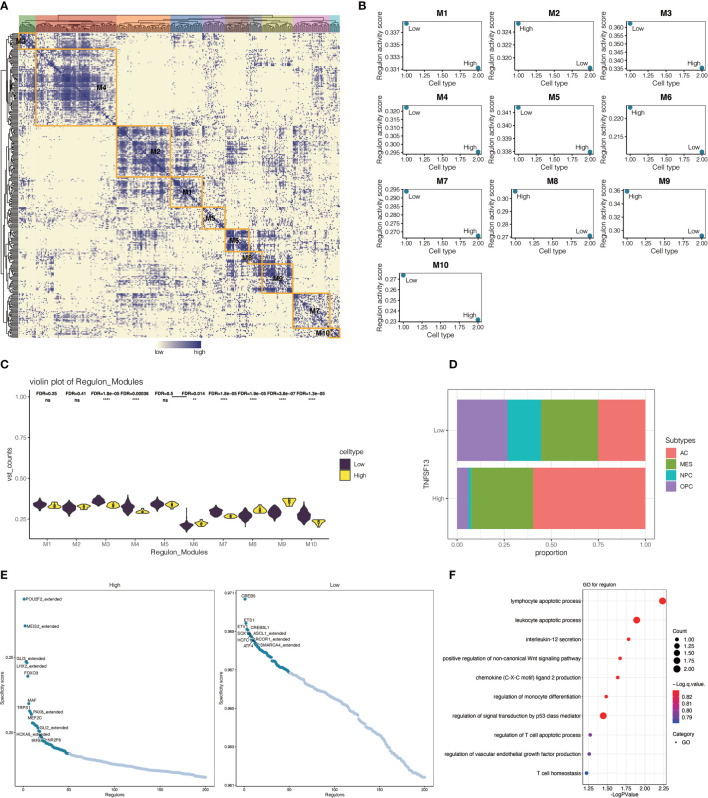
Interrelationship between transcription factors in malignant cells. **(A)** Heatmap of nine modules of transcription factors in malignant neoplastic cells. **(B)** Regulon activity scores of TNFSF13^high^ and TNFSF13^low^ malignant cells in nine modules based on transcription factors. **(C)** Violin plot for the distribution of TNFSF13^high^ and TNFSF13^low^ malignant cells in nine modules based on transcription factors. **(D)** Proportion of high and low TNFSF13 expression malignant neoplastic cells in four cellular states. **(E)** Scatter plot for the specificity score of transcription factors in high and low TNFSF13 expression malignant cells. **(F)** GO enrichment analysis based on high expression regulons in malignant cells. ns, not statistically significant; **P < .01, ****P < .0001.

In view of the crucial biological function of transcription factors in cells within tumor microenvironment, we performed a systematic gene regulatory network to excavate the mutual relation among transcription factors of malignant cells. On the basis of connection specificity index, malignant cells were clustered into nine main modules (**Figure 8A**), among which regulons were observed more gathering in module one and module three. Then we explored the interrelation between regulon distribution and TNFSF13 expression in neoplastic cells of all nine modules (**Figures 8B, C**), and the result showed high TNFSF13 samples appeared more regulon activity and counts in module three, module six, module seven, and module nine, while low TNFSF13 group appeared more regulon activity and counts in other five modules. Top specificity regulons in high TNFSF13 expression neoplastic cells were found including POU2F2_extended, MEIS2_extended, GLI3_extended, and LHX2_extended, while top regulons in low TNFSF13 neoplastic cells included CREB5, ETS1, ETV1, CREB3L1 (**Figure 8E**). Subsequently, we conducted GO enrichment analysis (**Figure 8F**) based on these top ranked regulons of both groups, and the result revealed a high correlation between these regulons and tumorigenic processes. Expounded by Neftel (63), additionally, single-cell analysis could classify malignant cells of GBM into four subclasses according to the expression spectrum of neoplastic cells: astrocyte-like (AC-like), mesenchymal-like (MES-like), neural-progenitor-like (NPC-like), and oligodendrocyte-progenitor-like (OPC-like). Our analysis revealed that compared with low TNFSF13 expression neoplastic cells, high TNFSF13 group exhibited higher proportions of AC-like and MES-like malignant cells (**Figure 8D**).

## Discussion

Increasing evidences have demonstrated the considerable influence of TNFSF13 in immunological suppression and immune infiltration, and TNFSF13 as a potential diagnostic biomarker and clinical management target is attractive. Considering that the molecular features of TNFSF13 in gliomas still remain unclear, we performed large-scale and integrated bioinformatic analyses to characterize TNFSF13 in gliomas. Our analyses initially indicated that TNFSF13 expression level was greatly upregulated in high-grade gliomas based on 2016 WHO classification, especially in IDH-wildtype status GBM. Moreover, TNFSF13 was remarkably upregulated in CL and ME subtypes compared with other subtypes among gliomas, which immune checkpoint inhibitors and immunosuppressive cytokines were enriched in ME subtype to facilitate immunosuppression and tumor invasion (49, 64). TNFSF13 was also observed enriched in hyperplastic blood vessels, leading edge and peri-necrotic zone. Additionally, 17 methylation probes identified from TCGA exhibited remarkable negative association with expression of TNFSF13, which further confirmed the correlation between TNFSF13 and unfavorable prognosis. Besides, existing researches have clarified that the overexpression of TNFSF13 associates with more severe classification and worse prognosis in diverse cancers, including breast cancer (31), multiple myeloma (32), non-small-cell lung cancer (65), and pancreatic cancer (66). And our analyses minutely testified the interrelation between TNFSF13 overexpression and worse survival of GBM patients based on TCGA and CGGA datasets. Besides, TNFSF13 played actively in regulating lymphocyte survival, activation, and differentiation of lymphocyte to promote immunosuppression (32, 67), which would ultimately attract a more severe prognosis. Taken together, these findings proved that TNFSF13 was closely associated with tumorigenesis and tumor progression.

The in-depth mechanisms of TNFSF13 involved in tumorigenesis was further determined. We further observed that several oncogenic drivers were detected as high amplification genomic peaks in TNFSF13 high expression cohort, including EGFR, CDK4, and PIK3CA (68). In the meantime, tumor suppressors like CDKN2A and CDKN2B were observed as deletion peaks in TNFSF13 high expression cohort (38). In view of the contribution from genomic alternations and heterogeneity to tumor progression, tumor microenvironment transformation, and drug tolerance (68), we supposed our analysis provided evidence for the relevance between TNFSF13 overexpression and degree of malignancy in gliomas.

Previous researches have indicated the potential effect of TNFSF13 to induce immune cells apoptosis, immunosurveillance collapse, and tumor invasion (27–29, 64). Our study initially investigated the correlation between TNFSF13 and ESTIMATE scores, including immune score, stromal score, and ESTIMATE score, among which all of them were identified positively correlated with TNFSF13 expression. Afterwards an analysis in regard to tumor microenvironment ingredient suggested the positive correlation between TNFSF13 expression and diversified infiltrating immune and stromal cells, including NK cells, monocytes, TEM, MDSCs, Tregs, neutrophils, fibroblasts. Moreover, we analyzed macrophage infiltration and T cell infiltration in 40 solid cancers with different algorithms and evaluated 10 immunogram scores for gliomas with different TNFSF13 expression levels, and all outcome revealed a relatively close connection between TNFSF13 overexpression and M2 macrophages, MDSCs, both of which were evidenced suppressing immunity response (69). Additionally, GSVA analysis proved that TNFSF13 might contribute to the inhibition of T-cell based antitumor immune processes, and investigation on inflammation response suggested TNFSF13 expression was upregulated in macrophage activation, T-cell-related signaling transduction, and antigen-presenting cells, while negatively associated with B-lymphocyte-related metagenes. These findings verified the impact of TNFSF13 on suppressing immunity response in glioma microenvironment. In addition, as the vital source of immune regulation, our analysis in regard to checkpoints indicates TNFSF13 closely synergistic with PDCD1LG2, HAVCR2, PDCD1, CD80, CTLA4, CD274, IDO1, and CD276 in pan-gliomas, LGGs, GBMs, and pan-cancer. Additionally, a robust relationship was observed between TNFSF13 and MMR, MSI, TMB, all of which were reliable markers for immunotherapy responses. Existing evidences have indicated that management with combination of immune checkpoint inhibitors would lead to higher immune response rate, along with more favorable survival in melanoma and brain metastasis cases (16, 34). These results suggest TNFSF13 might be a promising marker for immunotherapy response.

Noteworthily, increasing evidence have attested the crucial biological function of tumor-associated macrophages (TAMs) in tumor deterioration and metastasis (70–73). Metabolites produced by malignant cells would lead to the M2 polarization of macrophages and generate multiple cytokines and angiogenic factors, and this process ends up inducing the progression and invasion of tumor (74, 75). Our primary analysis has correspondingly verified the positive relevance between TNFSF13 expression and macrophage activation. Furthermore, single-cell RNA analysis was conducted to dig more potential pertinence of TNFSF13 in TAMs-related immunity and glioma deteriorate. The significance of scRNA-seq analysis on exploring gene function and immune microenvironment has been proved (76, 77). Among all scRNA-seq analysis content, pseudotime trajectories analysis was conducted to capture alterations on transcriptional level in the progression of tumor cells and macrophages (40). Our current study with scRNA-seq analysis testified that TNFSF13 was notably upregulated in M2 macrophages, T cells, and neoplastic cells, while pseudotime trajectories analysis and functional annotations for neoplastic cells and M2 macrophages forcefully proved that TNFSF13 expression was associated with macrophage infiltration and a series of tumorigenic signaling pathways, such as HIF-1 signaling pathway, canonical Wnt signaling pathway, and p53 signaling pathway. These results further validated the vital role of TNFSF13 in regulating macrophage activity and immune response signaling pathways. Notably, ligand-receptor interactions among different cell types showed that multiple ligand-receptor interactions existed among TNFSF13^high^ neoplastic cells, T cells, and macrophages, and these interactions involved cytokines, growth factors, checkpoints, and others. Of all factors detected, IL-8 has been proved conducive for cancer cells to escape immunoreaction through angiogenesis induced by macrophages (78, 79). C3 is a key member of complement system and promotes tumor progression by suppressing immune system as well (80). CD44 peculiarly impacts glioma outcomes in a biphasic way: high- and low-level CD44 expression both correlate with favorable survival, while intermedia CD44 level do not (81). Referred to existing researches, our analysis indicated that complicated ligand-receptor interactions worked within tumor microenvironment to regulate glioma malignancy and patient outcome, and expression of those factors was highly relevant to TNFSF13.

Diverse transcription factors were proved to be associated with glioma progression. Agminated transcription factor ETS1 would induce angiogenesis and neoplastic cell migration to contribute to tumor progression through upregulating KIF14 expression (82). HIF-1α and AHR modulate the metabolism of neoplastic and immune cells to impact tumor immunity and progression (83). Here, we continued scRNA-seq analysis and conducted systematic gene regulatory network to explore the interactions among transcription factors of malignant cells. Noteworthily, GO and KEGG enrichment analysis for those particular regulons mightily suggested the pertinence between these transcription factors and glioma malignancy, while TNFSF13 expression also correlated with regulon distribution.

Taken together, our elaborate bioinformatics analyses illuminated a potential influence of TNFSF13 in the progression and targeted anticancer treatment for human gliomas. TNFSF13 overexpression was particularly relevant to a high degree of gliomas malignancy and more severe prognosis in glioma cases. The significant correlation between TNFSF13 and inflammation, immunosuppression was verified. TNFSF13 was involved in the activation of M2 macrophage and potentially regulated the cell communication *via* IL-8, C3, CD44. TNFSF13 also mediated the activities of transcription factors including FOXO3, MEIS2, and IRF8. However, there are still limitation and deficiency in our study, existing data for the upregulation and activation of TNFSF13 to modulate the immune system still remains scarce, and we expected to improve that in subsequent researches. For instance, the correlation between TNFSF13 overexpression and B cell activation still remains unclear, then some immunization activities and poor clinical outcomes are contradictory to each other among high TNFSF13 patients. Further *in vivo* and *in vitro* researches on the regulatory mechanism of TNFSF13 for immunoregulation and tumor microenvironment in gliomas are expected.

## Code Availability

All statistical analysis was performed using R project. All codes can be inquired from the corresponding author.

## Data Availability Statement

The original contributions presented in the study are included in the article/**Supplementary Material**. Further inquiries can be directed to the corresponding authors.

## Author Contributions

Conception and design: HZ, XW, RC, and QC. Foundation support: QC and ZL. Acquisition and analysis of data: HZ, WW, XW, ZD, ZW, ZH, and XZ. Interpretation of data: HZ and XW. Drafting the manuscript and revising for submission quality: HZ and XW. Study supervision: QC. All authors contributed to the article and approved the submitted version.

## Funding

This work was supported by the National Natural Science Foundation of China (NO. 82073893, NO. 81703622, NO. 81873635), China Postdoctoral Science Foundation (NO. 2018M633002), Hunan Provincial Natural Science Foundation of China (NO. 2018JJ3838, NO. 2019JJ80056, NO. 2018SK2101), Hunan Provincial Health Committee Foundation of China (C2019186), Xiangya Hospital Central South University postdoctoral foundation, and Fundamental Research Funds for the Central Universities of Central South University.

## Conflict of Interest

The authors declare that the research was conducted in the absence of any commercial or financial relationships that could be construed as a potential conflict of interest.

## Publisher’s Note

All claims expressed in this article are solely those of the authors and do not necessarily represent those of their affiliated organizations, or those of the publisher, the editors and the reviewers. Any product that may be evaluated in this article, or claim that may be made by its manufacturer, is not guaranteed or endorsed by the publisher.
