# Supplementary material for: TNFSF13 Is a Novel Onco-Inflammatory Marker and Correlates With Immune Infiltration in Gliomas
Source: Front Immunol. 2021 Oct 12;12:713757. doi: 10.3389/fimmu.2021.713757 (PMC8546343; doi:10.3389/fimmu.2021.713757)
Supplement: Supplementary file 1 [file DataSheet_1.docx]

**Figure Legends**

**
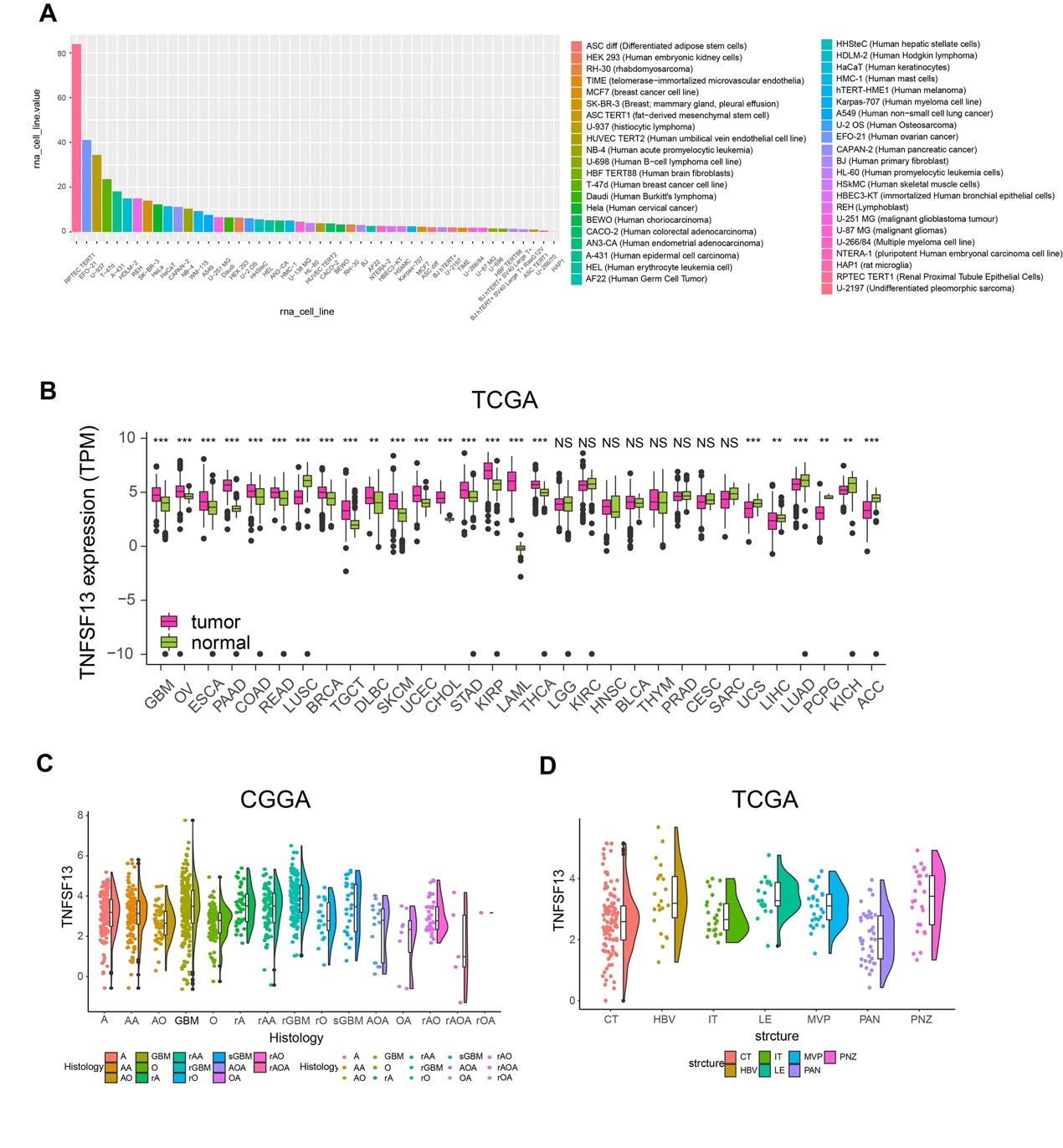
**

**Supplementary Figure 1. Clinical and molecular features in associations with TNFSF13 expression.** **(A)** TNFSF13 expression in various cell lines. **(B)** TNFSF13 expression in different tumors from TCGA dataset. GBM, Glioblastoma multiforme; OV, Ovarian serous cystadenocarcinoma; ESCA, Esophageal carcinoma; PAAD, Pancreatic adenocarcinoma; COAD, Colon adenocarcinoma; READ, Rectum adenocarcinoma; LUSC, Lung squamous cell carcinoma; BRCA, Breast invasive carcinoma; TGCT, Testicular Germ Cell Tumors; DLBC, Lymphoid Neoplasm Diffuse Large B-cell Lymphoma; SKCM, Skin Cutaneous Melanoma; UCEC, Uterine Corpus Endometrial Carcinoma; CHOL, Cholangiocarcinoma; STAD, Stomach adenocarcinoma; KIRP, Kidney renal papillary cell carcinoma; LAML, Acute Myeloid Leukemia; THCA, Thyroid carcinoma; LGG, Brain Lower Grade Glioma; KIRC, Kidney renal clear cell carcinoma; HNSC, Head and Neck squamous cell carcinoma; BLCA, Bladder Urothelial Carcinoma; THYM, Thymoma; PRAD, Prostate adenocarcinoma; CESC, Cervical squamous cell carcinoma and endocervical adenocarcinoma; SARC, Sarcoma; UCS, Uterine Carcinosarcoma; LIHC, Liver hepatocellular carcinoma; LUAD, Lung adenocarcinoma; PCPG, Pheochromocytoma and Paraganglioma; KICH, Kidney Chromophobe; ACC, Adrenocortical carcinoma. **(C)** TNFSF13 expression in different histopathologic classification from CGGA dataset. A, low-grade astrocytoma; AA, anaplastic astrocytoma; AO, anaplastic oligodendroglioma; GBM, glioblastoma; O, oligodendroglioma; rA, recurrent low-grade astrocytoma; rAA, recurrent anaplastic astrocytoma; rGBM, recurrent glioblastoma; rO, recurrent, oligodendroglioma; sGBM, secondary glioblastoma; AOA, anaplastic oligoastrocytoma; OA, oligoastrocytoma. **(D)** Intra-tumor analysis of PDIA3 expression. CT (Cellular Tumour), HBV (Hyperplastic Blood Vessels), IT (Infiltrating Tumor), LE (Leading Edge), MVP (Microvascular Proliferation), PAN (Pseudopalisading Cells Around Necrosis) and PNZ (Perinecrotic Zone). NS, *, **, and *** indicate p < .05, P < .01, p < .001, and no significant difference, respectively.

**
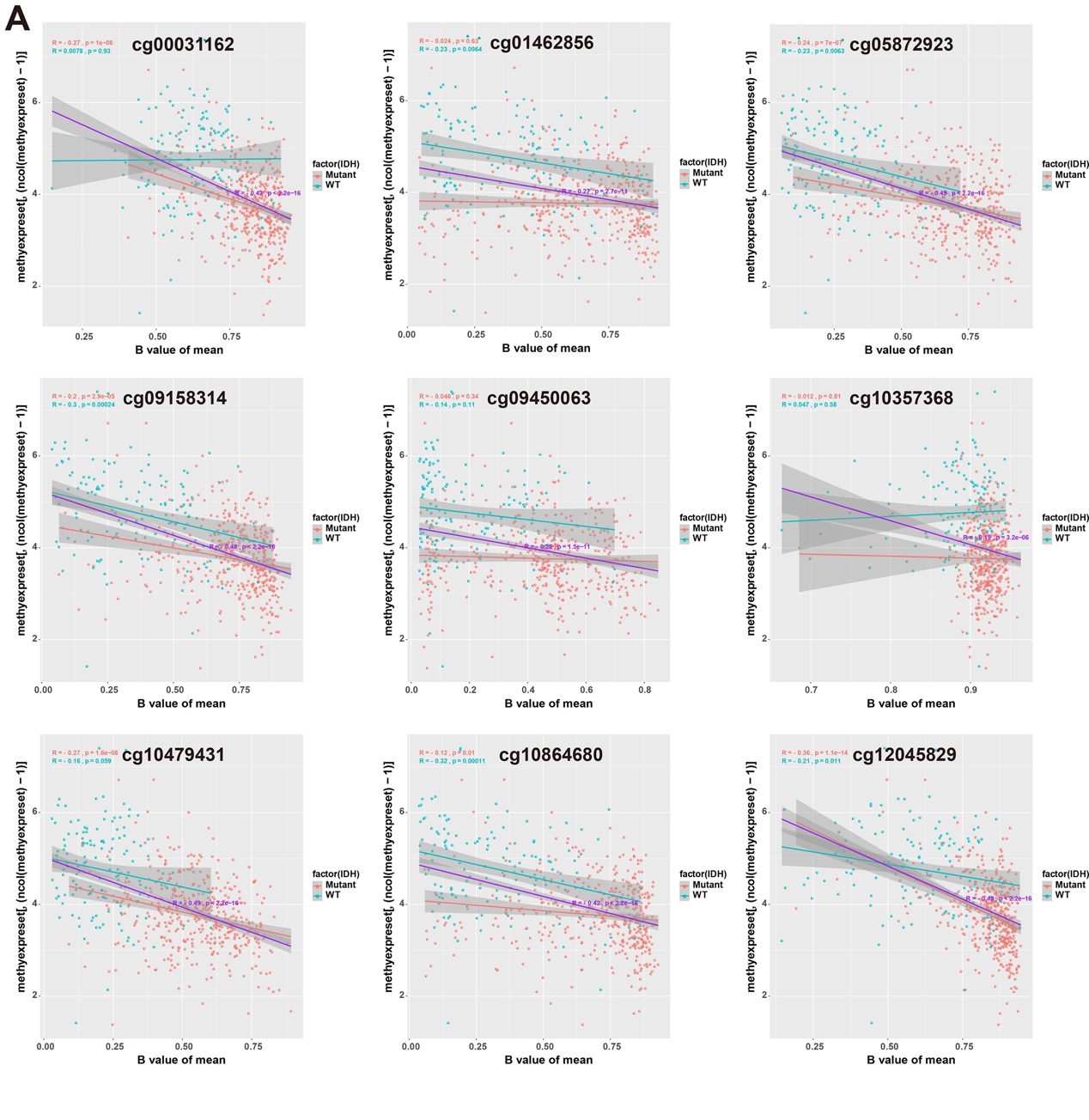
**

**Supplementary Figure 2. Regulation of TNFSF13 by methylation. (A)** Relationship between TNFSF13 and methylation status at promoter region in TCGA: cg00031162, cg01462856, cg05872923, cg09158314, cg09450063，cg10357368, cg10479431, cg10864680, cg12045829. The orange dots represent IDH‐mutant samples, and cyan dots represent IDH wild‐type samples, respectively. The orange line and cyan line represent linear regression between APOBEC3B expression and promoter region methylation in IDH‐mutant samples and IDH wild‐type samples, respectively.

**
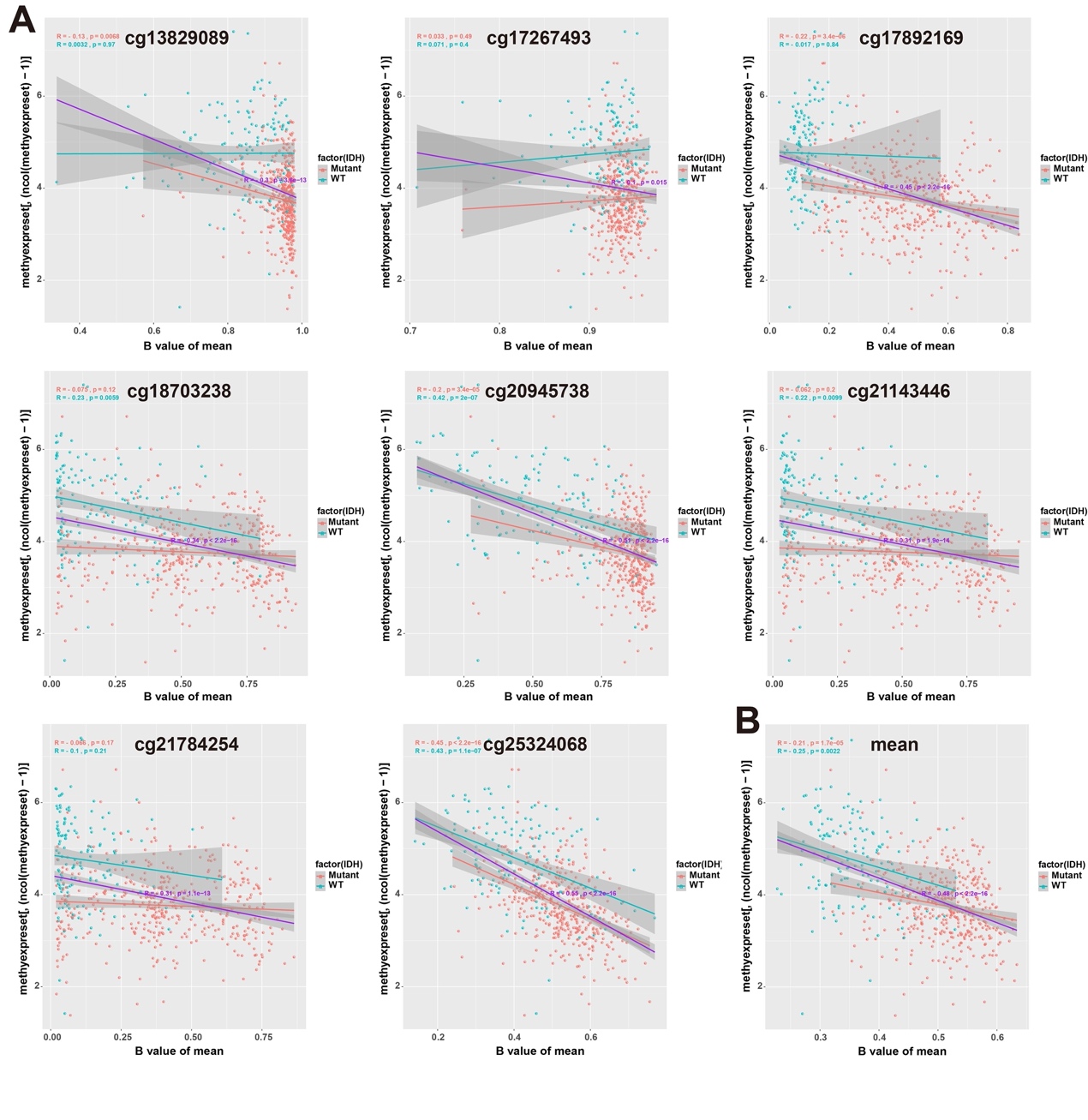
**

**Supplementary Figure 3. Regulation of TNFSF13 by methylation. (A)** Relationship between TNFSF13 and methylation status at promoter region in TCGA: cg13829089，cg17267493，cg17892169，cg18703238，cg20945738，cg21143446, cg21784254, cg25324068. The orange dots represent IDH‐mutant samples, and cyan dots represent IDH wild‐type samples, respectively. The orange line and cyan line represent linear regression between APOBEC3B expression and promoter region methylation in IDH‐mutant samples and IDH wild‐type samples, respectively. **(B)** Relationship between TNFSF13 and the mean value of methylation status at promoter region in TCGA.

**
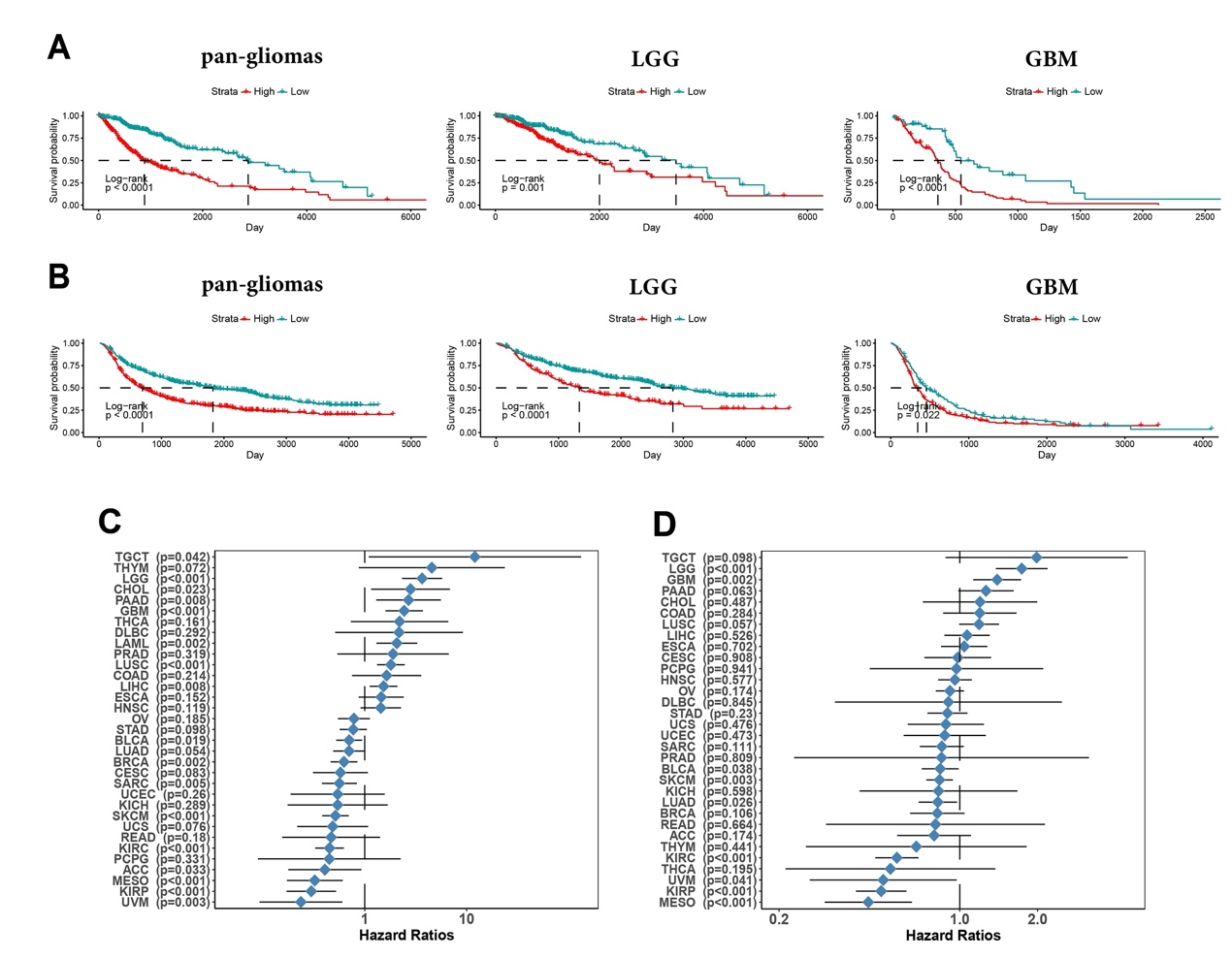
**

**Supplementary Figure 4. TNFSF13 expression is associated with overall survival (OS) in glioma patients.** Kaplan-Meier analysis of OS was performed based on high and low expression of TNFSF13 in pan-glioma, LGG and GBM patients from **(A)** TCGA and **(B)** CGGA datasets. **(C)** Subgroup analyses assessing prognostic value of TNFSF13 in OS in various tumor types from TCGA. **(D)** Subgroup analyses assessing prognostic value of TNFSF13 in DSS in various tumor types from TCGA. The length of horizontal lines indicates the 95% confidence interval of each group. The vertical dotted line indicates the Hazard Ratios (HR) of all patients. HR < 1.0 means high TME score is a favorable prognostic biomarker. TGCT, Testicular Germ Cell Tumors; THYM, Thymoma; LGG, Brain Lower Grade Glioma; CHOL, Cholangiocarcinoma; PAAD, Pancreatic adenocarcinoma; GBM, Glioblastoma multiforme; THCA, Thyroid carcinoma; DLBC, Lymphoid Neoplasm Diffuse Large B-cell Lymphoma; LAML, Acute Myeloid Leukemia; PRAD, Prostate adenocarcinoma; LUSC, Lungsquamous cell carcinoma; COAD, Colon adenocarcinoma; LIHC, Liver hepatocellular carcinoma; ESCA, Esophageal carcinoma; HNSC, Head and Neck squamous cell carcinoma; OV, Ovarian serous cystadenocarcinoma; STAD, Stomach adenocarcinoma; BLCA, Bladder Urothelial Carcinoma; LUAD, Lung adenocarcinoma; BRCA, Breast invasive carcinoma; CESC, Cervical squamous cell carcinoma and endocervical adenocarcinoma; SARC, Sarcoma; UCEC, Uterine Corpus Endometrial Carcinoma; KICH, Kidney Chromophobe; SKCM, Skin Cutaneous Melanoma; UCS, Uterine Carcinosarcoma; READ, Rectum adenocarcinoma; KIRC, Kidney renal clear cell carcinoma; PCPG, Pheochromocytoma and Paraganglioma; ACC, Adrenocortical carcinoma; MESO, Mesothelioma; KIRP, Kidney renal papillary cell carcinoma;UVM, Uveal Melanoma. P-values were obtained from the log-rank test.

**
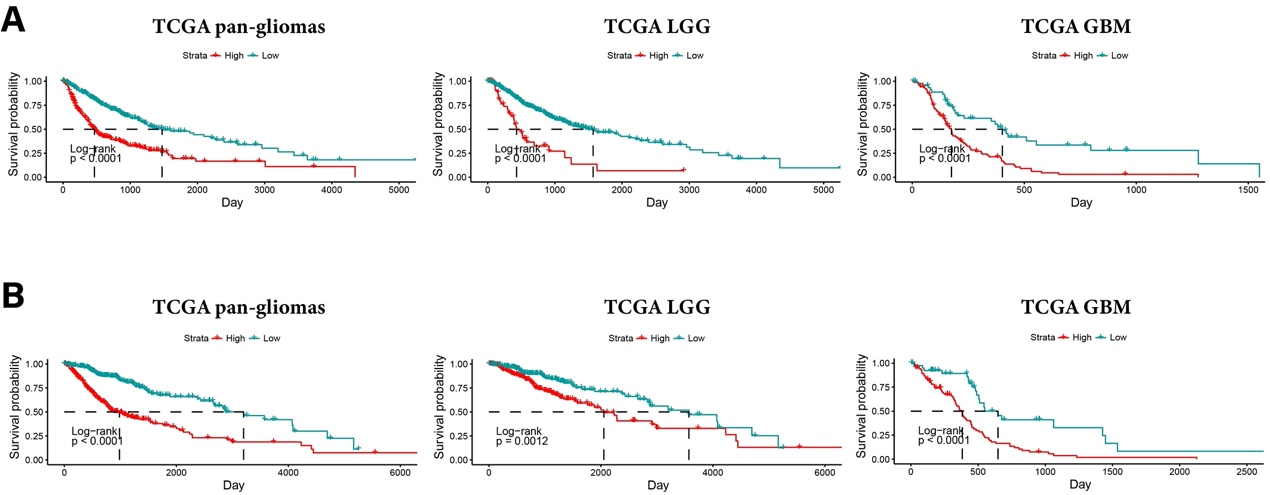
**

**Supplementary Figure 5**. Kaplan-Meier analysis using high vs low TNFSF13 expression for **(A)** progression-free survival (PFS) in pan-glioma, LGG and GBM patients in TCGA dataset and **(B)** disease specific survival (DSS) in pan-glioma, LGG and GBM patients in TCGA dataset. *P*-values were obtained from the log-rank test.


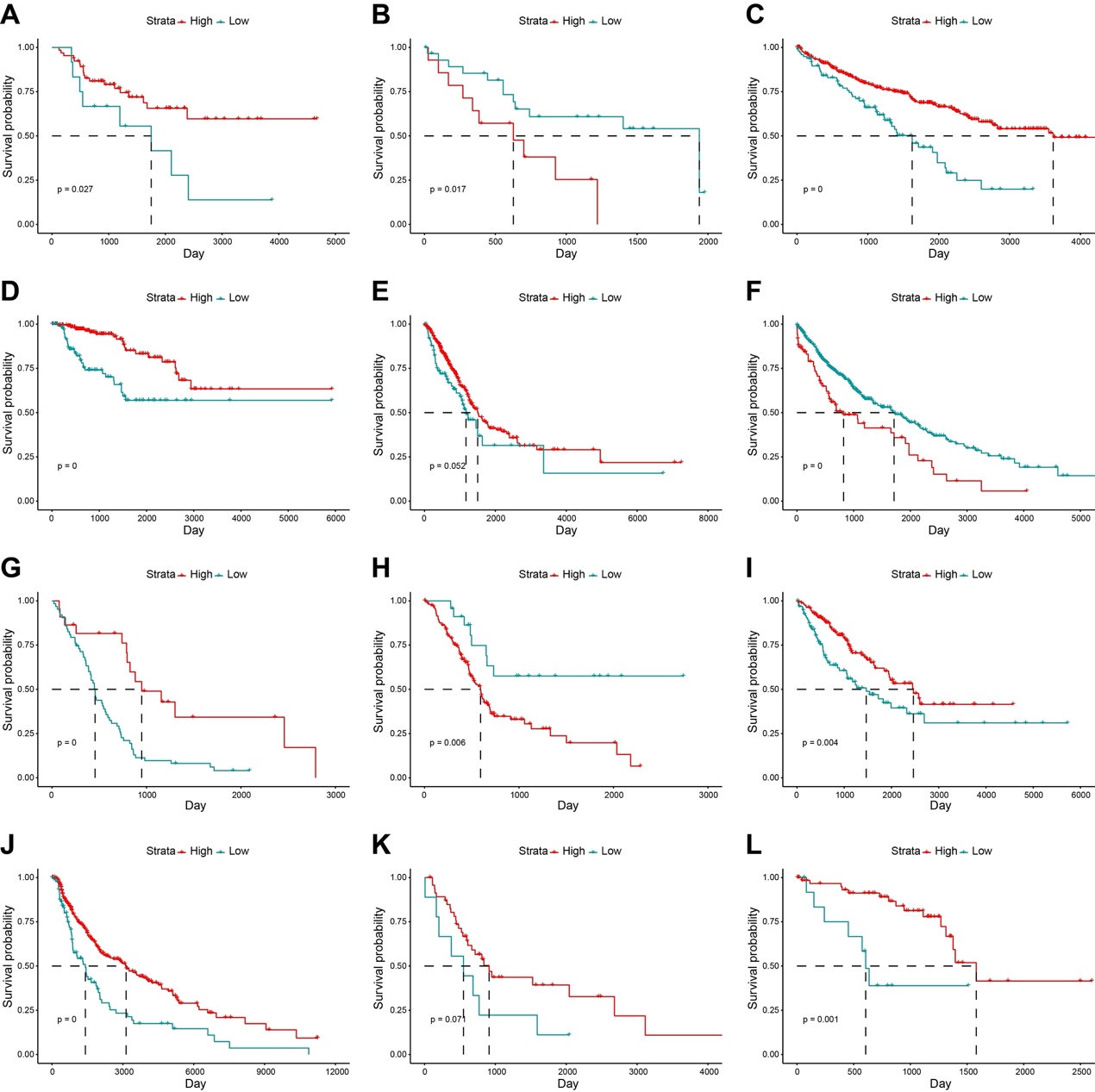


**Supplementary Figure 6. Kaplan-Meier analysis for disease specific survival (DSS) based on high vs low TNFSF13 expression in various tumors. (A)** ACC, Adrenocortical carcinoma; **(B)** CHOL, Cholangiocarcinoma; **(C)** KIRC, Kidney renal clear cell carcinoma; **(D)** KIRP, Kidney renal papillary cell carcinoma; **(E)** LUAD, Lung adenocarcinoma; **(F)** LUSC, Lung squamous cell carcinoma; **(G)** MESO, Mesothelioma; **(H)** PAAD, Pancreatic adenocarcinoma; **(I)** SARC, Sarcoma; **(J)** SKCM, Skin Cutaneous Melanoma; **(K)** UCS, Uterine Carcinosarcoma; **(L)** UVM, Uveal Melanoma. *P*-values were obtained from the log-rank test.

**
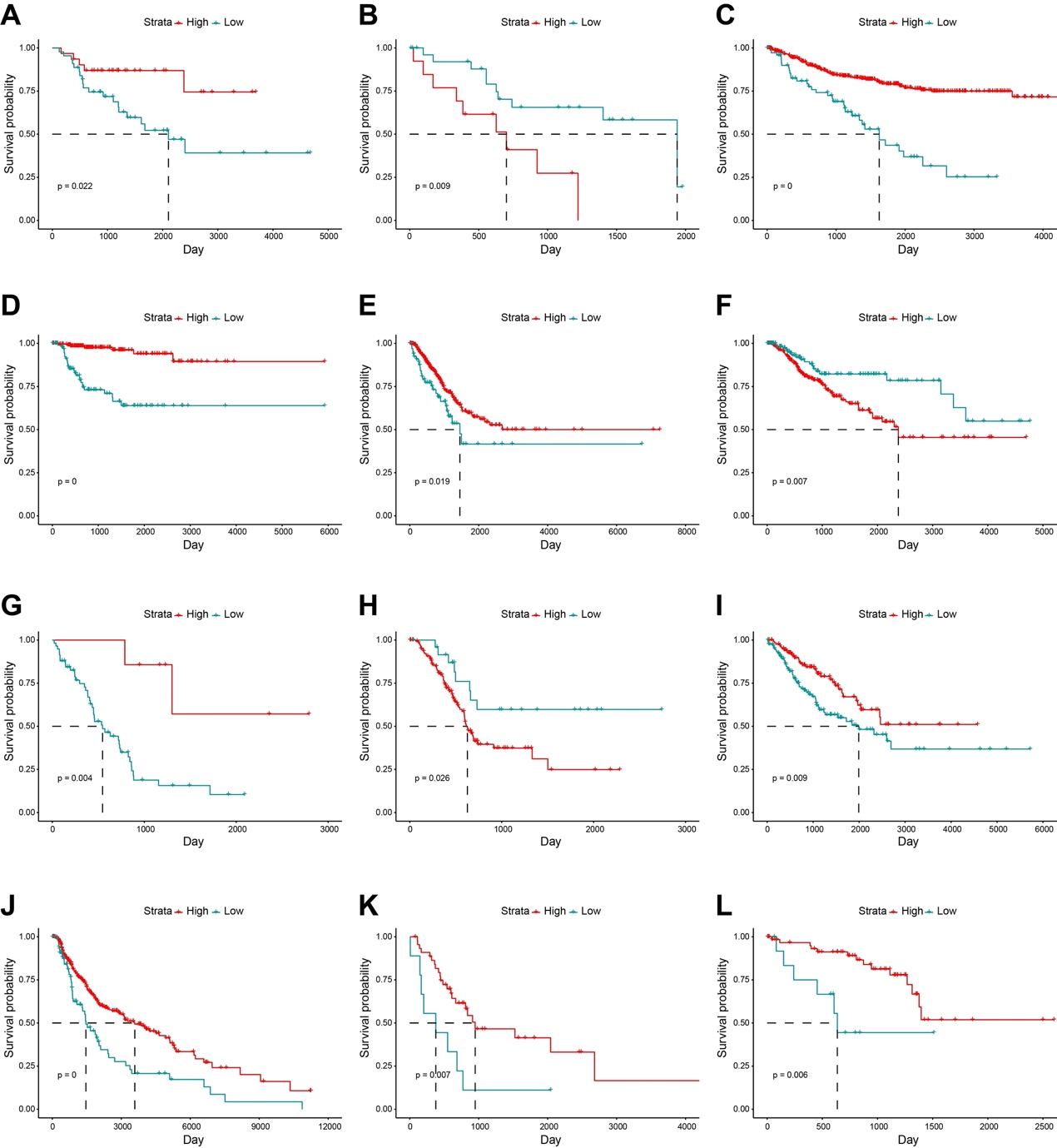
**

**Supplementary Figure 7. Kaplan-Meier analysis for overall survival (OS) based on high vs low TNFSF13 expression in various tumors. (A)** ACC, Adrenocortical carcinoma; **(B)** CHOL, Cholangiocarcinoma; **(C)** KIRC, Kidney renal clear cell carcinoma; **(D)** KIRP, Kidney renal papillary cell carcinoma; **(E)** LUAD, Lung adenocarcinoma; **(F)** LUSC, Lung squamous cell carcinoma; **(G)** MESO, Mesothelioma; **(H)** PAAD, Pancreatic adenocarcinoma; **(I)** SARC, Sarcoma; **(J)** SKCM, Skin Cutaneous Melanoma; **(K)** UCS, Uterine Carcinosarcoma; **(L)** UVM, Uveal Melanoma. *P*-values were obtained from the log-rank test.


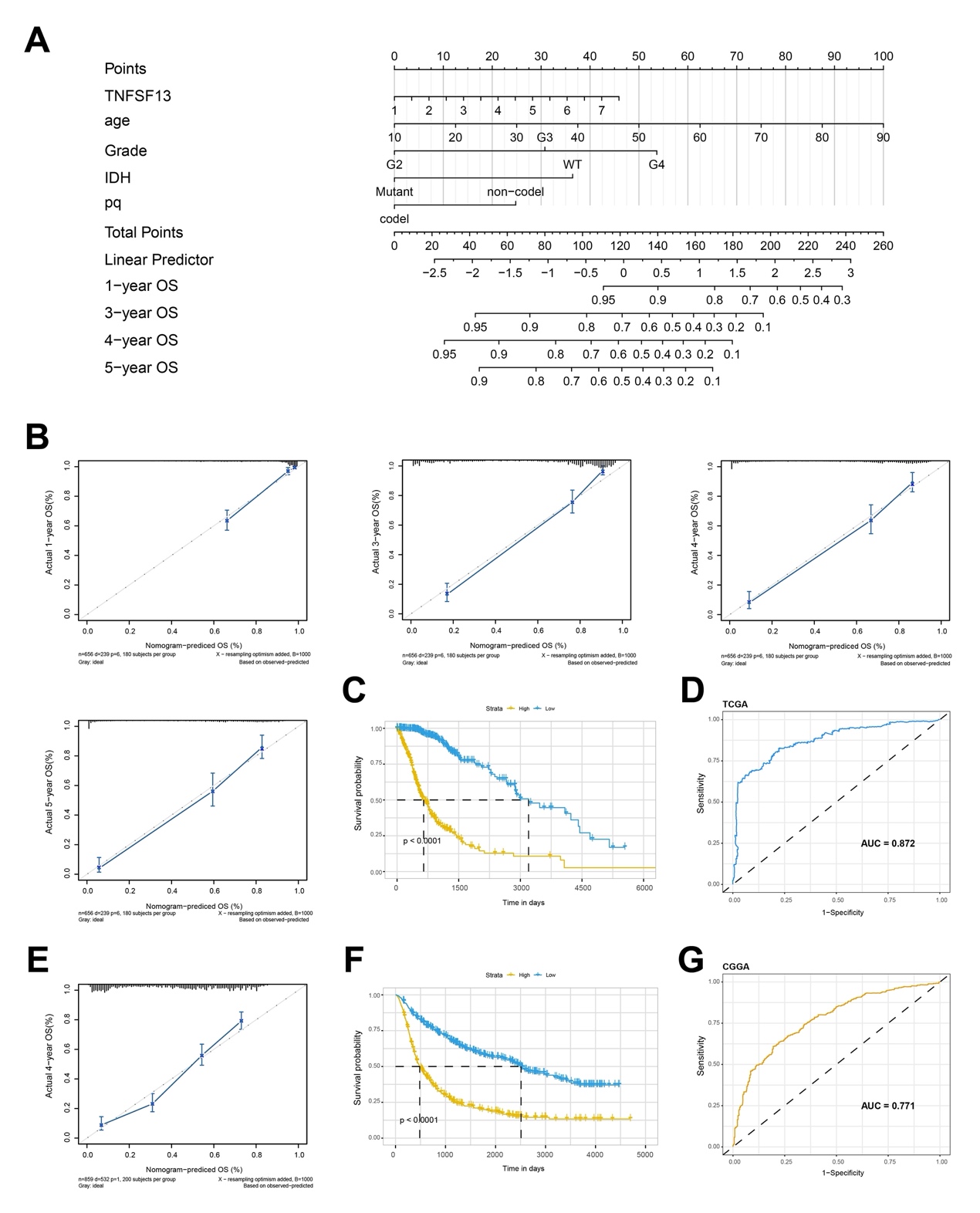


**Supplementary Figure 8. TNFSF13 expression is relevant to poor survival outcome in glioma patients. (A)** Nomogram to predict clinical prognosis for glioma patients with OS and five independent risk factors (the expression level of TNFSF13, age, grades of gliomas, mutation of IDH and 1p19q codeletion). Calibration plots Based on clinical 1-year OS, 3-year OS, 4-year OS and 5-year OS in **(B)** TCGA and **(E)** CGGA datasets. Kaplan-Meier analysis for overall survival (OS) based on high vs low risk patients in **(C)** TCGA and **(F)** CGGA datasets. Receiver operating characteristic (ROC) curve showed 87.2% and 77.1% sensitivity and specificity of TNFSF13 to predict survival outcome of gliomas patients in **(D)** TCGA and **(G)** CGGA datasets.


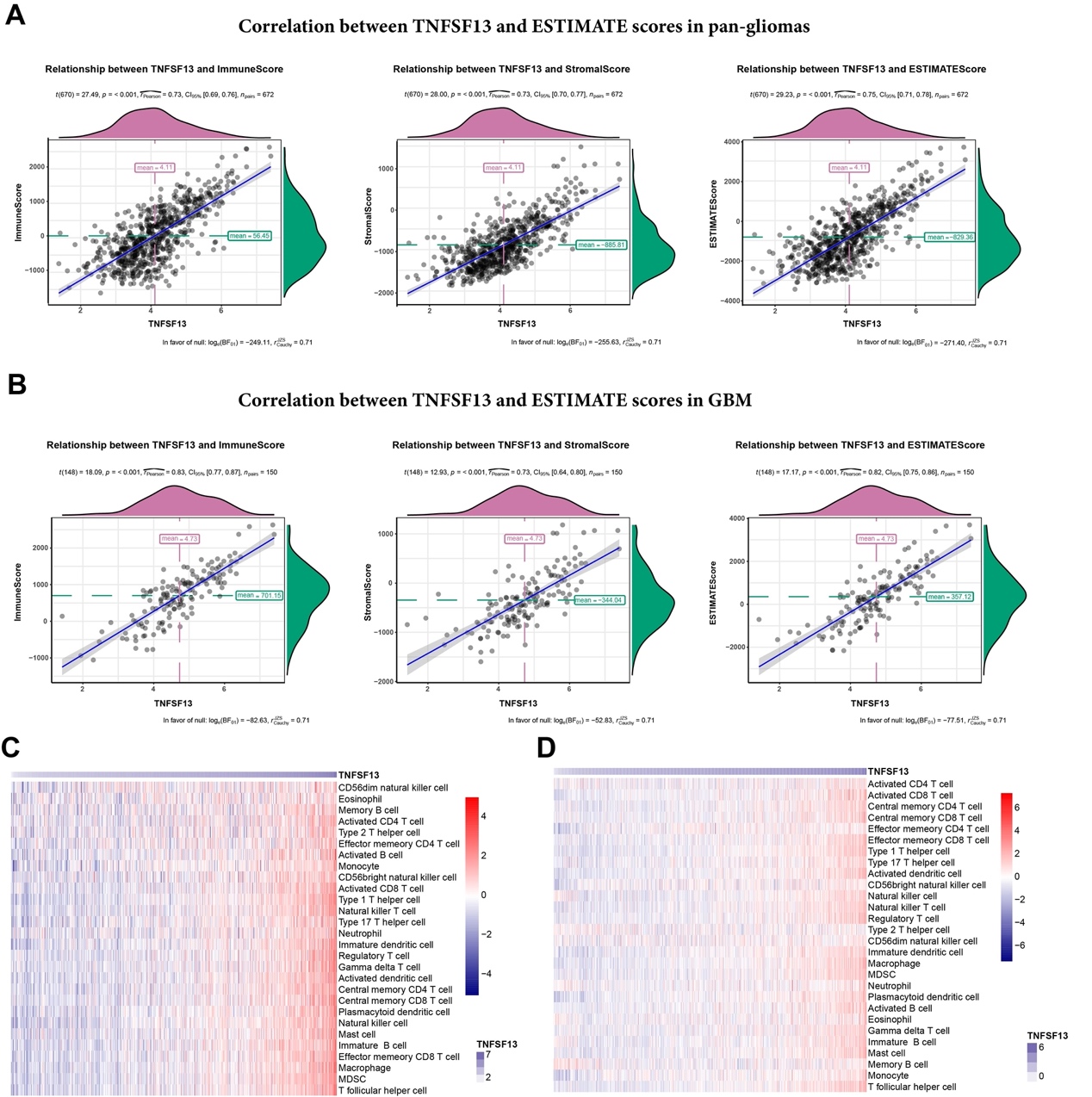


**Supplementary Figure 9. TNFSF13 expression in correlation with ESTIMATE scores in gliomas.** TNFSF13 expression was correlated with increasing immune score, stromal score and ESTIMATE score in **(A)** pan-glioma and **(B)**. GBM patients. Heatmaps indicate TNFSF13-associated specific infiltrating cell types according to **(C)**. TCGA and **(D)**. CGGA pan-glioma datasets.

**
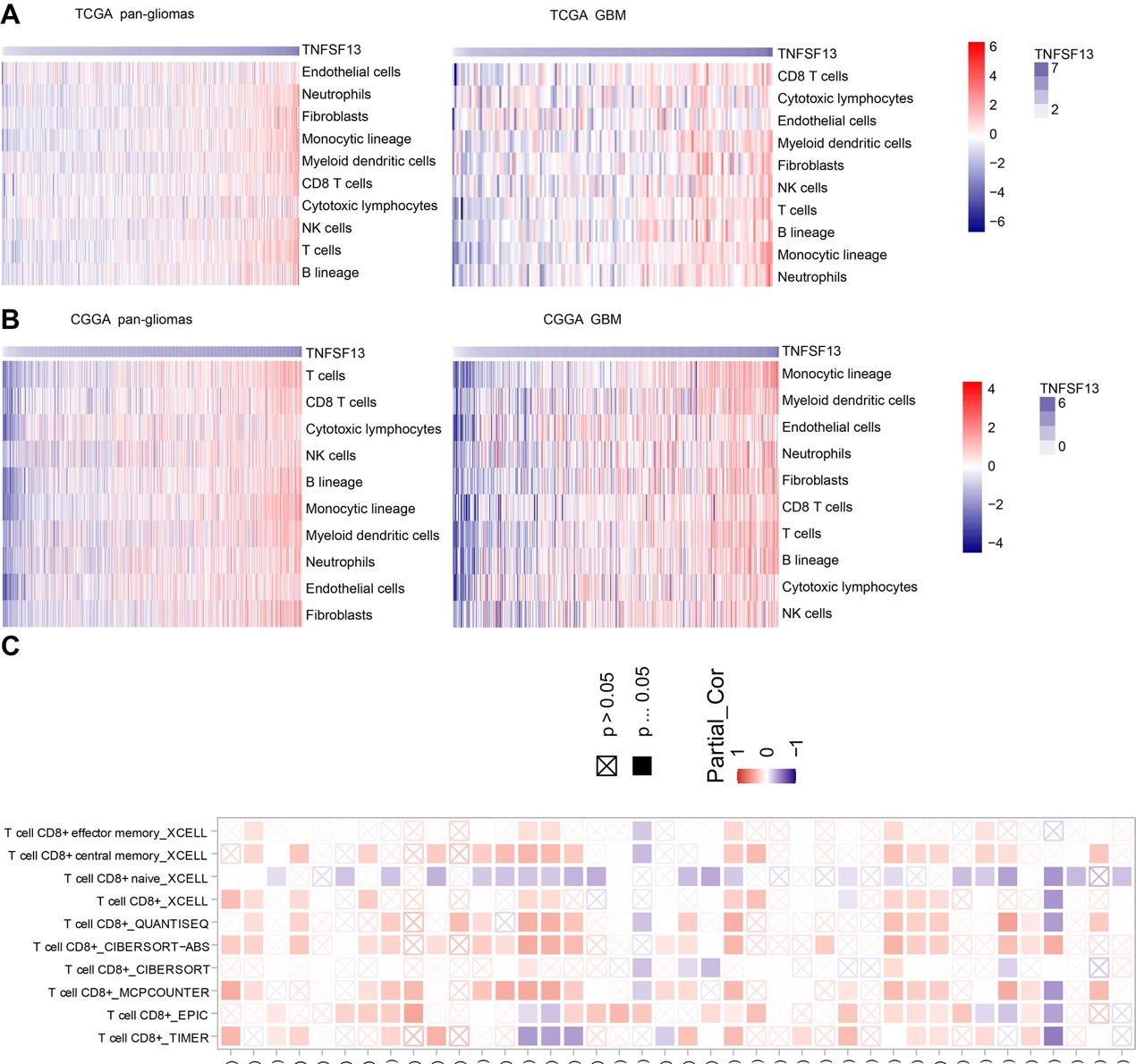
**

**Supplementary Figure 10.** Relationship between TNFSF13 and 10-immune cell lineages genes among pan-glioma and GBM cases from **(A)** TCGA and **(B)** CGGA datasets. Z-transformed expression values are colored in red for high expression and blue for low expression, as denoted in the scale bar. **(C)** Immune infiltration assessment for pan cancer samples regarding T cell based on TIMER 2.0, red represents high expression of T cell, purple represents low expression of T cell.


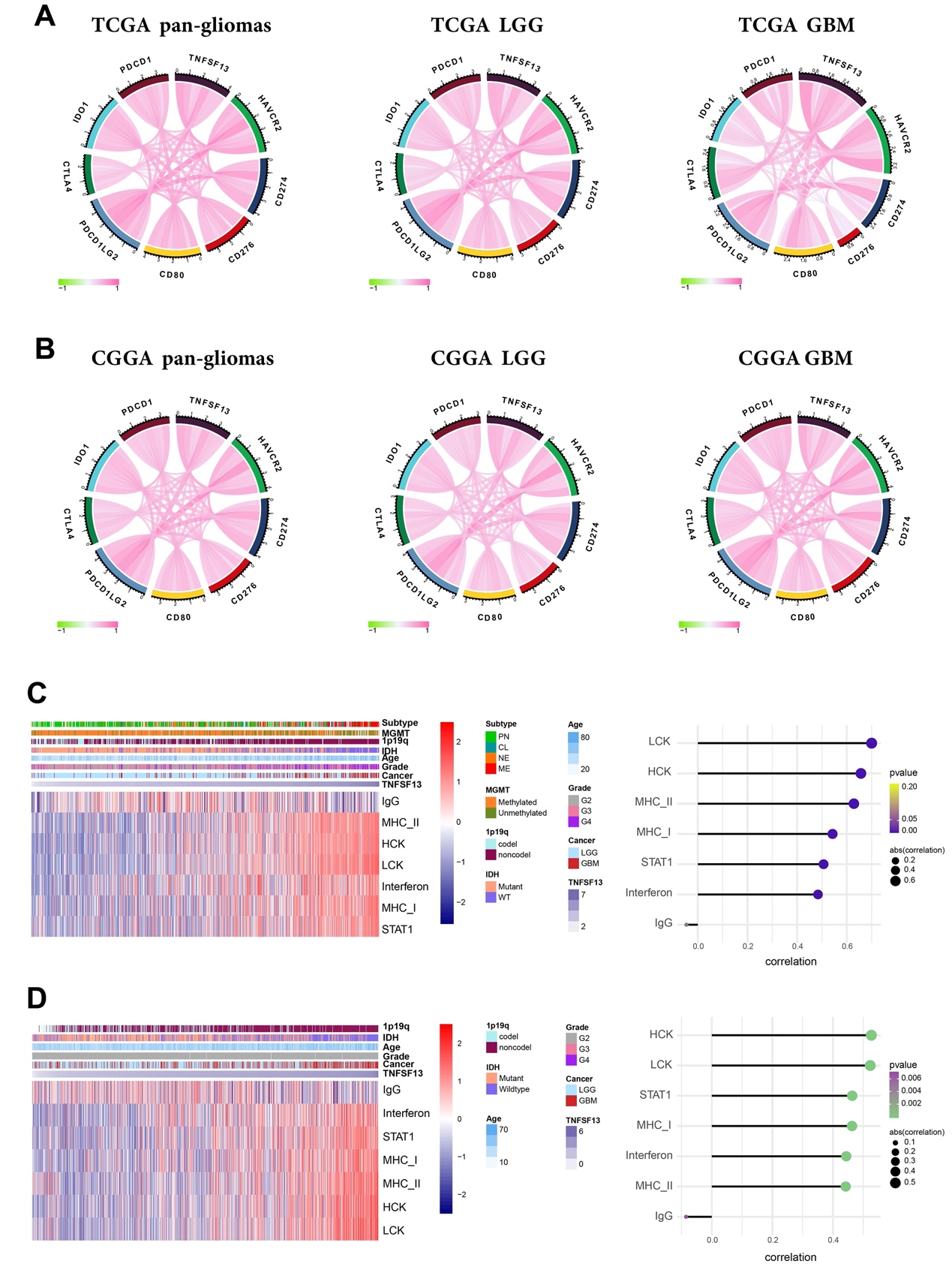


**Supplementary Figure 11. Association between TNFSF13 and immune checkpoint members and inflammatory activities.** Correlation between TNFSF13 and other immune checkpoint molecules in pan-gliomas, LGG, and GBM from **(A)** TCGA and **(B)** CGGA datasets. Heatmaps illustrate the relationship between TNFSF13 and inflammatory activities in pan gliomas from **(C)** TCGA and **(D)** CGGA dataset.


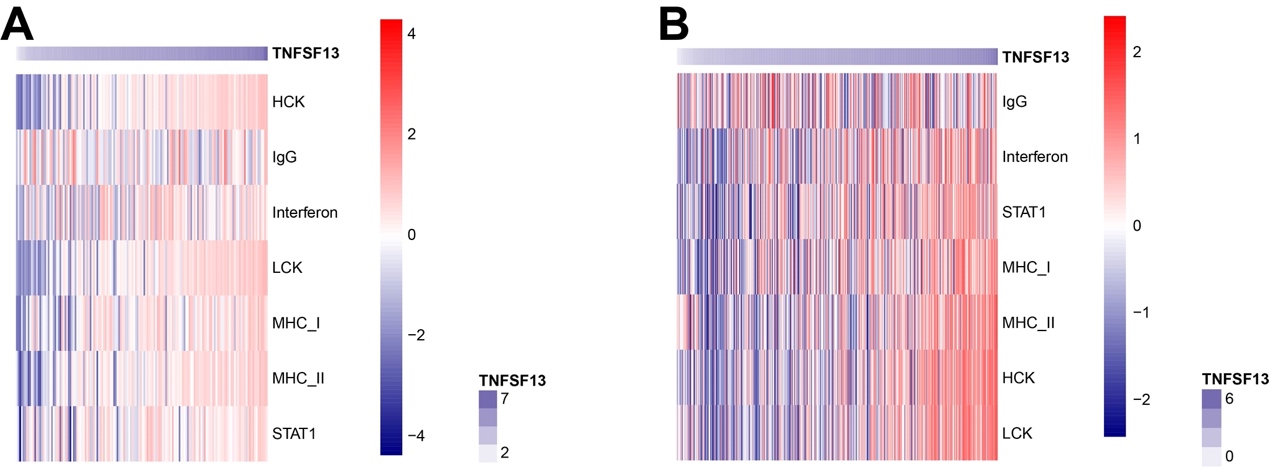


**Supplementary Figure 12.** Correlation between TNFSF13 expression and inflammatory metagenes among GBM cases in **(A)** TCGA and **(B)** CGGA datasets. Z-transformed expression values are colored in red for high expression and blue for low expression, as denoted in the scale bar.


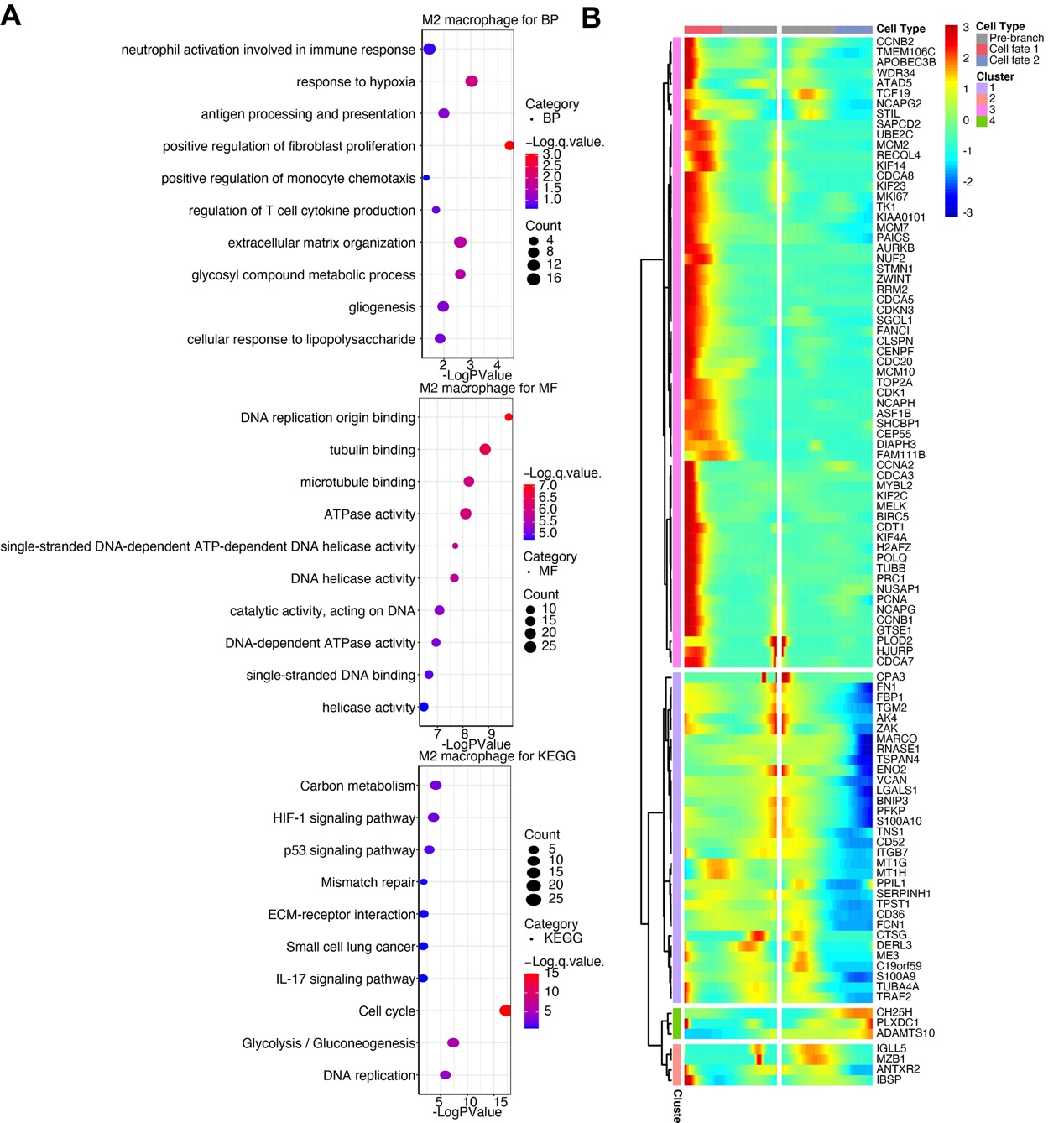


**Supplementary Figure 13. Differentially expressed genes (DEGs) and functional annotations of M2 macrophages in single-cell pseudotime trajectories. (A)** Biological processes (BP) and molecular functions (MF) from GO enrichment analysis and KEGG pathway analysis based on DEGs for branch point 1 in macrophages. **(B)** Heatmap of top 100 DEGs with branch-dependent expression for branch point 1 in macrophages.


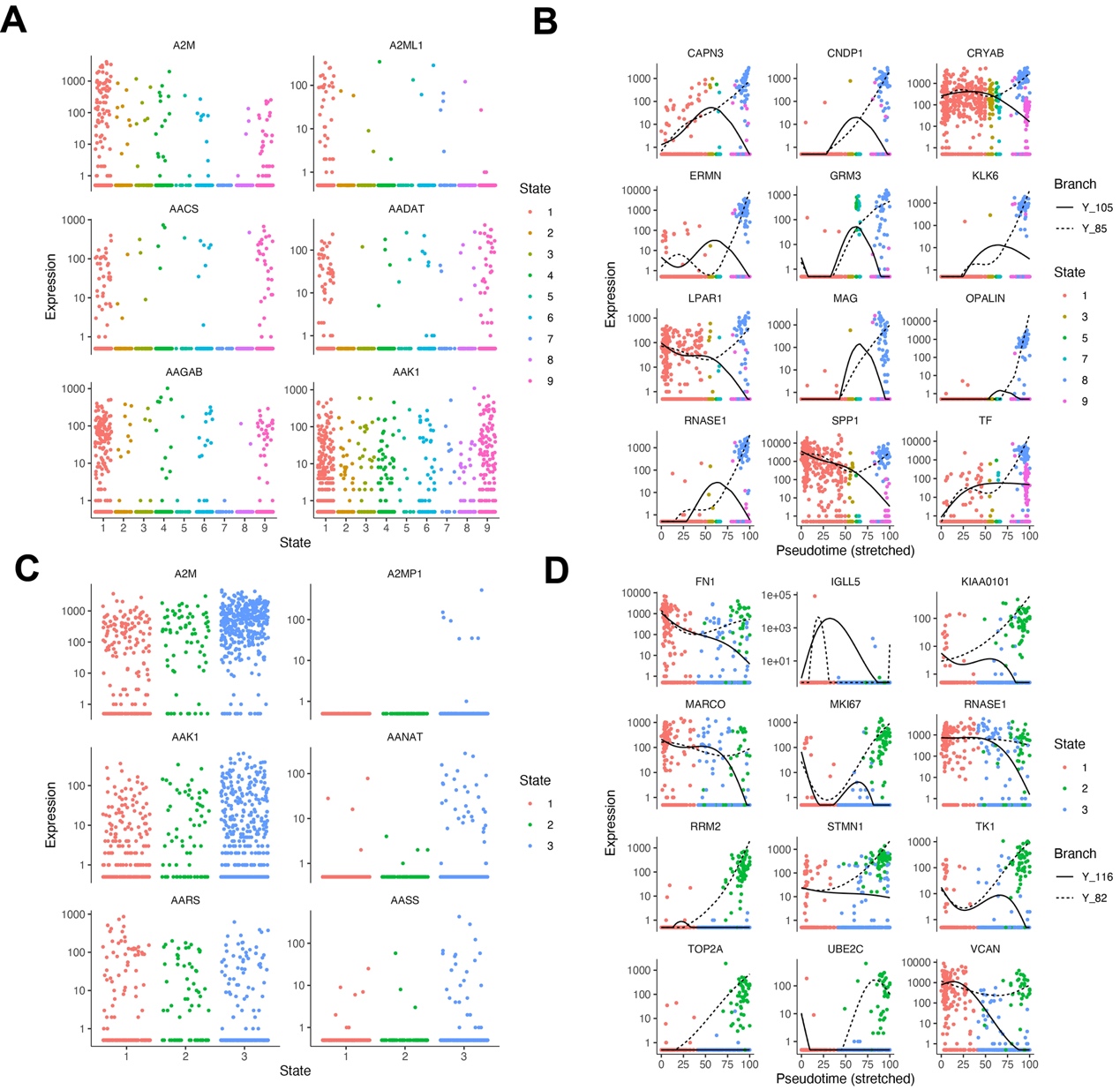


**Supplementary Figure 14. Single-cell pseudotime trajectories of neoplastic cells and M2 macrophages in GBM.** **(A)** Top 6 differentially expressed genes among 5 states in pseudotime trajectories of neoplastic cells. **(B)** Top 12 genes with branch-dependent expression for branch point 2 in neoplastic cells. **(C)** Top 6 differentially expressed genes among 5 states in pseudotime trajectories of macrophages. **(D)** Top 12 genes with branch-dependent expression for branch point 1 in macrophages.

**Table S1. Clinical characteristics of Xiangya cohort**
